# StrEAMM-Thioether: Efficient Structure Prediction for Thioether-Linked Cyclic Peptides

**DOI:** 10.1021/acs.jpcb.5c06368

**Published:** 2026-02-06

**Authors:** Minh Ngoc Ho, Jiayuan Miao, Yi Shan, Choi Yi Li, Hiroaki Suga, James D. Baleja, Yu-Shan Lin

**Affiliations:** † Department of Chemistry, School of Arts and Sciences, 1810 Tufts University, Medford, Massachusetts 02155, United States; ‡ Department of Developmental, Molecular, & Chemical Biology, Pharmacology and Drug Development Program, Graduate School of Biomedical Sciences, School of Medicine, Tufts University, Boston, Massachusetts 02111, United States; § Department of Chemistry, Graduate School of Science, 98341 The University of Tokyo, Tokyo 113-0033, Japan

## Abstract

Cyclic peptides have gained interest as potential therapeutics due to their ability to target specific protein–protein interactions and be membrane-permeable. Understanding the sequence–structure relationship of cyclic peptides would greatly benefit their rational design. However, cyclic peptides tend to adopt multiple conformations in solution, and it remains challenging to use experimental techniques such as solution NMR to delineate their structural ensembles: i.e., the different structures a cyclic peptide adopts and the associated populations. Alternatively, molecular dynamics (MD) simulations can be used to provide such information. However, MD simulations are computationally expensive and not applicable for large-scale screening. Our group has developed the StrEAMM (Structural Ensembles Achieved by Molecular Dynamics and Machine Learning) computational platform and applied it to predict structural ensembles of head-to-tail cyclized pentapeptides and hexapeptides. However, head-to-tail cyclized peptides can be challenging to synthesize due to low yield and complicated reaction workup and product isolation. Furthermore, head-to-tail cyclized peptides are not compatible with screening techniques like mRNA display. Here, we expand the StrEAMM method to thioether-linked cyclic peptides, a popular scaffold in mRNA display. The trained graph neural network models are able to provide fast and simulation-quality structural ensembles for thioether-linked cyclic peptides. Using these models, we identify four thioether-linked cyclic pentapeptides that are predicted to be the best-structured and subsequently experimentally synthesize and characterize them by solution NMR. We observe general agreement between the predicted structures and the NMR results. Ultimately, we envision that StrEAMM-thioether models can work synergistically with the current mRNA platform to streamline the resource-intensive process of drug discovery and design of cyclic peptides.

## Graphical abstract

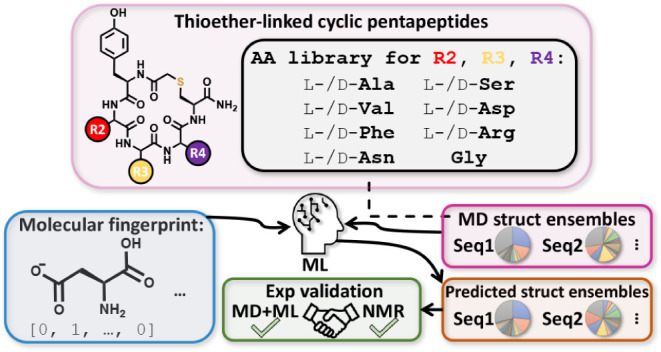

## Introduction

Protein–protein interactions (PPIs) are involved in many biological processes, and their dysregulation is linked to various diseases.^1–3^ The ability to target specific PPIs has great therapeutic potential. Unfortunately, while membrane-permeable small molecules can target, for example, enzyme binding pockets well, their small surface areas make them insufficient to effectively interfere with PPIs.^4,5^ On the other hand, biologics like antibodies have large surfaces and can thus effectively modulate PPIs. However, biologics are large proteins and not membrane-permeable, thus typically not orally bioavailable and unable to reach intracellular targets.^6^

Cyclic peptides have shown great applications and promise in disrupting PPIs.^7,8^ However, most approved cyclic peptides are natural products or derived from natural products,^9^ and it remains difficult to develop cyclic peptide drugs that can target a specific protein of interest.^10^ For example, while drug design can greatly benefit from structural information, using NMR to characterize the solution structures of cyclic peptides is generally difficult because they tend to adopt multiple conformations.^11–13^ On the other hand, cyclic peptide structures obtained by X-ray crystallography are stabilized by crystal packing and may not necessarily reflect complete solution structural ensembles. Therefore, there is currently limited structural information on cyclic peptides, which significantly hampers their *de novo* rational design.

Molecular dynamics (MD) simulations have become a valuable tool for studying the structures of cyclic peptides.^11,14–16^ However, MD simulations are computationally expensive and not applicable for large-scale screening. Realizing the demand for high-throughput screening for cyclic peptide structures, our group has successfully developed the StrEAMM platform (Structural Ensembles Achieved by Molecular Dynamics and Machine Learning) to enable rapid, simulation-quality structure prediction of head-to-tail cyclized pentapeptides and hexapeptides in <1 s per sequence.^17,18^ In brief, our group performed bias-exchange metadynamics (BE-META) simulations for the cyclic peptide sequences in the training data sets, obtained their structures and the corresponding populations, and used them as training instances for linear regression, convolutional neural network (CNN), and graph neural network (GNN) models.

These current StrEAMM models represent a significant leap toward the efficient design of cyclic peptides because they can be used to, for example, identify cyclic peptide binders that adopt the desired structures for downstream synthesis. Nonetheless, synthesizing head-to-tail cyclized peptides is challenging due to low yield^19–21^ and complicated reaction workup and product isolation.^22^ Another class of cyclic peptides is thioether-linked peptides, which possess a relatively more straightforward cyclization chemistry and are commonly used in screening techniques such as mRNA displays.^23,24^ While there are different ways to create thioether-linked cyclic scaffolds,^23^ a popular scaffold used in mRNA displays, particularly in the RaPID (Random nonstandard Peptides Integrated Discovery) system, is where the thioether bond is formed via a nucleophilic attack by the thiol group of the downstream-elongating Cys and the *N*-chloroacetyl electrophile group of the initiating amino acid (Figure 1).^25–28^ Disulfide bond formation is also a common method for peptide cyclization used in mRNA display.^29,30^ However, this chemical bond is more susceptible to cleavage under reductive conditions than the thioether bond,^31–34^ and losing the conformational constraints of designed cyclic therapeutics can lead to a loss of desired action.^35^ Therefore, the use of a more stable thioether bond presents a better alternative when screening for cyclic peptides.

**Figure 1:**
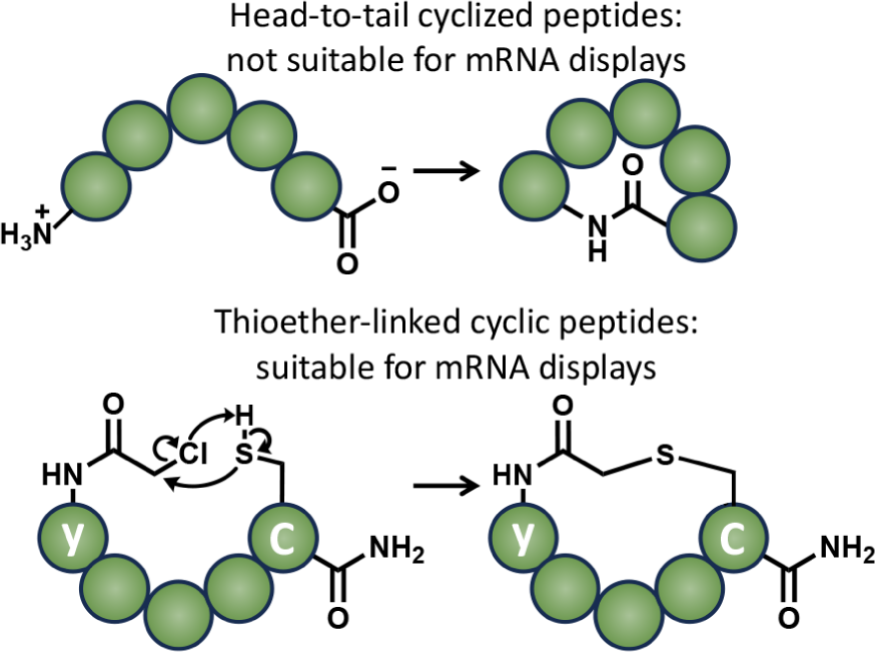
Illustrations of head-to-tail cyclized peptides and the thioether-linked cyclic peptide scaffold used in this study. Amide formation between the N- and C-termini is a common approach for cyclizing linear peptides, but this approach is not compatible with screening platforms such as mRNA displays. On the other hand, cyclizing a linear peptide via thioether formation between the N-terminus and the side chain of a Cys is suitable for mRNA displays. The shown scaffold with a d-Tyr at the N-terminus is a popular choice in mRNA displays, among many others.^23–28^

Besides the convenient cyclization chemistry, thioether-linked cyclic peptides can offer different conformational spaces synergistically with head-to-tail cyclic peptide structures to aid the drug design process. Furthermore, thioether-linked cyclic peptides and their derivatives—modified by substituting the thiol group to mitigate oxidation at the thioether bond—have been reported as potential drug candidates for treating cancer (via PD-L1 inhibition),^36^ high cholesterol (targeting PCSK9),^37–39^ and other conditions.^40,41^

Inspired by the high therapeutic potential of thioether-linked cyclic peptides, a machine learning (ML) framework would be beneficial to provide efficient and simulation-quality structural ensembles for this class of cyclic peptides. In this work, we expand the StrEAMM application to thioether-linked cyclic pentapeptides as a proof-of-concept study (Figure 2). A structure of a thioether-linked cyclic peptide was described by discretizing the backbone dihedral angles of each amino acid within a sequence and the thioether-linker-related dihedral angles using structural binning maps (Figure 3) in a similar fashion to our previous StrEAMM models for head-to-tail cyclic peptides.^17,18^

**Figure 2:**
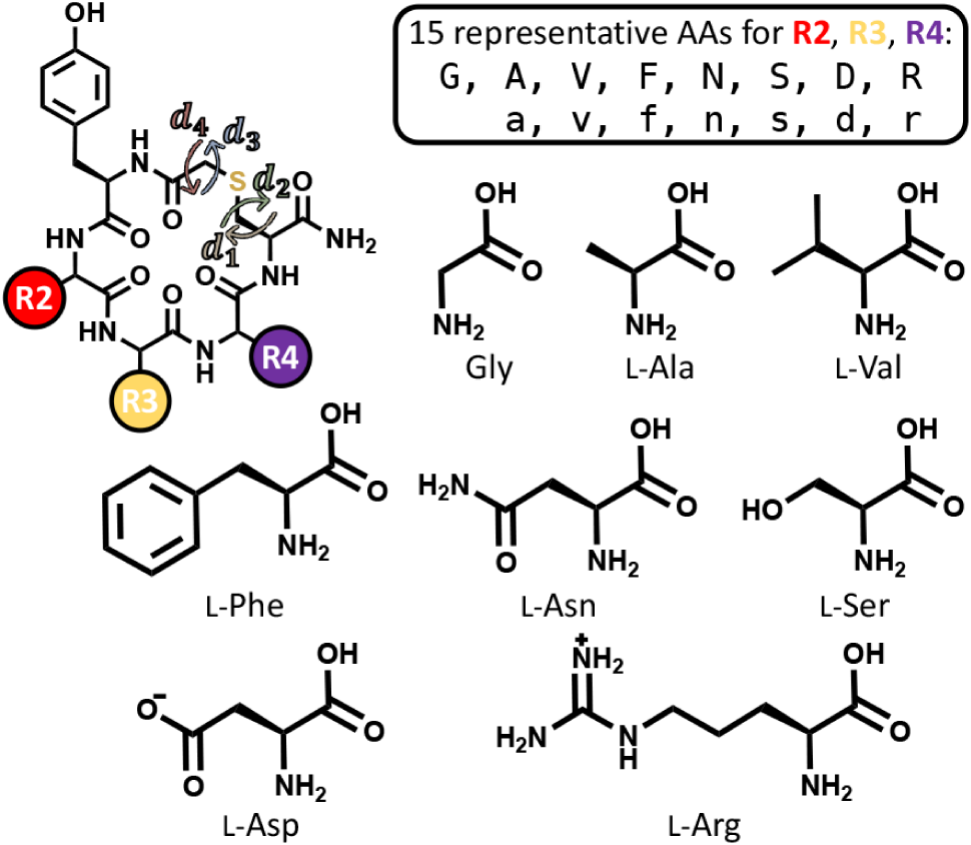
Thioether-linked cyclic pentapeptide scaffold used in this work. The sulfur atom of the Cys residue, which cyclizes the peptide sequence, is dark yellow. A 15-amino-acid (15-aa) library is used in the training, validation, and test data sets for the StrEAMM-thioether models. The lowercase letters denote d-amino acids. For brevity, chemical structures of only Gly and amino acids in l-forms are shown, but their mirror images are also included in the library. The four dihedral angles *d*_1_–*d*_4_ of the thioether linkage are labeled. See Figure 3 for atoms in each torsion.

**Figure 3:**
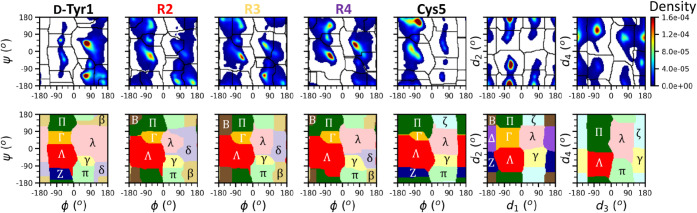
Structural binning maps for the thioether-linked cyclic pentapeptide scaffold derived from the dihedral density distributions of five amino acids in their (φ, ψ) spaces as well as from the linker dihedral density distribution to describe the linker orientation using all 396 training sequences. The four dihedral angles characterizing the linker orientation are the following: *d*_1_ ( $N_{i}$ – $C_{\alpha_{i}}$ – $C_{\beta_{i}}$ – $S_{\gamma_{i}}$ ); *d*_2_ ( $C_{\alpha_{i}}$ – $C_{\beta_{i}}$ – $S_{\gamma_{i}}$ – $C_{\delta_{i}}$ ); *d*_3_ ( $C_{\beta_{i}}$ – $S_{\gamma_{i}}$ – $C_{\delta_{i}}$ – $C_{\epsilon_{i}}$ ); *d*_4_ ( $S_{\gamma_{i}}$ – $C_{\delta_{i}}$ – $C_{\epsilon_{i}}$ – $N_{i+1}$ ), where *i* and *i*+1 denote the current and next residues, respectively. Structural digits are denoted using Greek letters.

In addition, we apply graph neural networks (GNNs) to explore structural ensembles of all thioether-linked cyclic pentapeptide sequence space. We report four peptides that were predicted to be the best-structured. Solution NMR characterization confirms that the MD-predicted top structure is within the top five of the NMR-derived ensembles for each peptide. The application of the StrEAMM model can work alongside the current mRNA display platform to speed up drug discovery and design of cyclic peptides.

## Materials and Methods

### Data Sets

In this study, we focus on thioether-linked cyclic pentapeptides. We generated diverse sequences, performed MD simulations with enhanced sampling, and analyzed the simulation results to obtain structural ensembles. The rationale for choosing the 5-amino-acid (5-aa) ring was 3-fold. First, there are therapeutically relevant cyclic peptides of size 5.^42–44^ Second, smaller cyclic peptides tend to be more membrane-permeable,^45^ which can be an important property when targeting intracellular targets or designing orally bioavailable drugs. Third, it is generally possible to obtain converged structural ensembles for this size of cyclic peptides with enhanced sampling.

The thioether-linked cyclic peptide scaffold used here (d-Tyr as the first amino acid and Cys as the last amino acid) was inspired from hits or their optimized versions from mRNA experiments.^25–27,44,46–48^ While these cyclic peptides have l-/d-Phe, l-/d-Tyr, or l-/d-Trp as the first amino acid, we chose d-Tyr for our thioether-linked cyclic pentapeptide scaffold because (1) Tyr is one of the versatile residues in modulating protein–protein interactions,^49^ and (2) d-amino acids can improve peptide proteolytic stability against enzymatic degradation.^50,51^

The amino acid library used to generate the thioether-linked cyclic pentapeptides contained G, A, V, F, N, S, D, R, a, v, f, n, s, d, and r, where lowercase letters denote d-amino acids. These 15 amino acids are a representative subset of the canonical amino acids and their d-forms: Gly is the smallest and achiral; Ala is the smallest chiral amino acid; Val has a β-branching side chain; Phe has an aromatic side chain; Asn has an amide-group side chain; Ser has a hydroxyl-group side chain; Asp has a negatively charged side chain; and Arg has a positively charged side chain (Figure 2).

The training data set included 396 sequences that were generated based on the following protocol: (1) 15 out of 396 sequences were randomly generated so that each of the three variable positions (R2, R3, and R4) can have all amino acids from the 15-aa library; (2) 43 out of 396 sequences were generated to have each amino acid from the 15-aa library at position R2, R3, or R4, while the residues at the other two variable positions were Gly (e.g., yAGGC, yGAGC, yGGAC, etc.); (3) the remaining training sequences were randomly generated with no duplication (List S1). For the test data set, 50 randomly generated sequences were created with no duplication (List S2).

### Charge Derivation for Thioether-Linked Fragment

To derive partial charges for the thioether-linked fragment, we used the tethered d-Tyr–Cys (via a thioether bond) fragment from the scaffold with an *N*-methyl (NME) cap at the C-termini of both d-Tyr and Cys and an acetyl (ACE) cap at the N-terminus of Cys (Figure S1). The initial structures used for charge derivation of this thioether-linked fragment were created from combinations of (φ, ψ) dihedral angles of Cys and d-Tyr adopting the α-helix or β-sheet backbone conformations and four randomized linker dihedral angles along the thioether linker (Figure 2). The (φ, ψ) values for Cys and Tyr, and also the dihedral values for the Tyr side chain, were taken from Cieplak et al.^52^ To generate the backbone and side chain angles of d-Tyr, we flipped those of Tyr to represent the mirror images. For each of the four backbone combinations of Cys and d-Tyr, 16 conformations were generated along the four linker dihedral angles. In total, 64 initial structures of the capped thioether-linked fragment were created using PyMOL.^53^ Steric clashes arising from the initial structures were energy-minimized by using the command "clean” with the MMFF94 force field in PyMOL.

The output structures from PyMOL’s “clean” command were geometry-optimized using the Gaussian16 (revision C.01) program with tight convergence criteria at the HF/6-31G­(d) level of theory.^54^ The (φ, ψ) dihedral angles of Cys and d-Tyr, as well as the side chain of d-Tyr, were frozen for each structure. For any structures that failed to converge after 100 optimization steps, they were rerun with an additional frozen χ_1_ angle on Cys. Similarly, an additional frozen χ_2_ angle on Cys was included when the geometry optimization of the additional frozen χ_1_ angle did not converge. Out of 64 structures of the capped thioether-linked fragment, seven structures across the four backbone combinations converged after freezing the χ_1_ angles, one structure from the β Cys and α d-Tyr combination converged after freezing both χ_1_ and χ_2_ angles, while all the others converged without the need of freezing side chain dihedrals.

The 64 optimized structures of capped thioether-linked fragments were submitted to the R.E.D. web server for charge derivation.^55^ The molecular electrostatic potential for each structure was computed using Gaussian16 with IOp (6/33 = 2, 6/41 = 4, 6/42 = 6) at the HF/6-31G­(d) level of theory, followed by a two-stage RESP^52,56^ charge fitting method to compute partial charges. Charges of the backbone amide N and H atoms, as well as those of the carbonyl C and O atoms, in Cys and d-Tyr of the thioether-linked fragment were assigned to be consistent with the charges for all the neutral canonical amino acids in the AMBER ff99SB force field.^57^ Because the ACE and NME caps were used to help mimic the peptide environment, charges of N and H atoms in the NME cap, and charges of C and O atoms in the ACE cap, were set to be consistent with those of the backbone atoms in Cys and d-Tyr of the thioether-linked fragment. Additionally, the net charges of ACE, NME, and d-Tyr were constrained to zero. The derived partial charges of Cys (containing the thioether bond and the carboxyl group linking to the NH of d-Tyr) were used to subsequently simulate thioether-linked cyclic peptides (Figure S1), while the partial charges of d-Tyr and the caps from these RESP fittings were discarded.

### Molecular Dynamics Simulations and Principal Component Analysis

To sample structural ensembles of the thioether-linked cyclic pentapeptides, we employed bias-exchange metadynamics (BE-META)^58^ simulations with the TIP3P water model and the residue-specific force field with CMAP potentials (RSFF2C).^59^ General Amber Force Field (GAFF) was also used for bonded parameters involving the thioether linker.^60^ BE-META simulations have been previously applied to study the conformational ensemble of cyclic peptides.^15,16,61,62^

For each BE-META simulation, the initial structure was solvated in a water box with a minimum distance of at least 1.0 nm between the atoms of the peptide and the walls of the box. The system net charge was neutralized with Na^+^ or Cl^–^ counterions. The system was subjected to energy minimization using the steepest descent algorithm, followed by two stages of equilibration. In the first stage, all heavy atoms of the peptide were position-restrained using a harmonic potential with a force constant of 1,000 kJ·mol^–1^·nm^–2^. In this stage, the system underwent a 50 ps simulation at 300 K in an NVT ensemble and a following 50 ps simulation at 300 K and 1 bar in an NPT ensemble. In the second stage, positional restraints were removed, and the system was equilibrated in the same NVT and NPT ensembles for 100 ps each. The production simulations were performed at 300 K and 1 bar in an NPT ensemble. The leapfrog algorithm with a time step of 2 fs was used to integrate the equations of motion. The bonds involving hydrogen atoms were constrained using the LINCS algorithm. The nonbonded interaction cutoff was set to 1.0 nm. For Coulombic interactions beyond the cutoff, particle mesh Ewald summation with a Fourier grid spacing of 0.12 nm and an order of 4 was used. Long-range van der Waals interactions were treated with dispersion corrections for energy and pressure. Temperature was controlled by velocity rescaling with a coupling time constant of 0.1 ps. Parrinello–Rahman barostat was used for pressure control with a coupling time constant of 2.0 ps and an isothermal compressibility of 4.5 × 10^–5^ bar^–1^.

The BE-META simulations were performed using GROMACS 2018.8 ^63^ and PLUMED 2.6.6 plugin.^64^ We employed two-dimensional (2D) biases of (φ_*i*_’, ψ_*i*_’), (ψ_*i*_’, φ_*i*+1_’), and torsional motions in the thioether linker to promote conformational sampling of this scaffold.^65,66^ φ_*i*_’ was defined by H–N–C_α_–C and ψ_*i*_’ was defined by N–C_α_–C–O, and they were used to reduce the probability of the formation of *cis* amide bonds due to the added biases. Five neutral replicas (i.e., replicas with no bias) were used to obtain unbiased structural ensembles for subsequent analysis.

To monitor and verify simulation convergence, two different initial structures were used for two parallel BE-META simulations per cyclic peptide sequence. For each thioether-linked cyclic pentapeptide, the two different initial structures (referred to as S1 and S2) had an RMSD ≥ 1.3 Å (calculated using the heavy atoms constituting the ring of a cyclic peptide, i.e., backbone N, C_α_, C atoms, and the atoms along the thioether linker). Dihedral principal component analysis (dPCA)^67^ was used to analyze the backbone dihedrals and the four linker dihedral angles along the thioether linker (Figure 2) and compute the density distributions in the projected 3D space of the first three principal components. Normalized integrated product (NIP)^68^ between two parallel simulations of each cyclic peptide was calculated in the 3D space from the top three principal components to monitor the convergence of the simulations. If the NIP between the two parallel simulations was ≥0.9, then the simulations were deemed converged. We used trajectories of 50–100 ns to compute the NIP. If the simulations did not converge, we extended them to 200 ns and calculated the NIP using trajectories of 100–200 ns to check for convergence. If the simulations still did not converge, we extended them to 300 ns and calculated the NIP using trajectories of 200–300 ns. If the simulation did not converge using 200–300 ns trajectories, we also calculated the NIP using 100–300 ns trajectories. Sequences that did not converge within 300 ns were discarded, and new sequences were substituted to ensure high-quality data. Besides MD simulations of the training and test data sets, we also simulated an additional set of cyclic peptides, which were used to compare with the ML model predictions to identify the best-structured cyclic peptides for experimental verification (Table 1). This data set contained 39 sequences, of which 8 were from the training set, and 31 were new. A summary of converged sequences within each interval and of sequences that failed to converge is shown in Table S1. Files describing the BE-META simulation setup can be found on GitHub (https://github.com/ysl-lab/CP_tutorial).

**Table 1:**
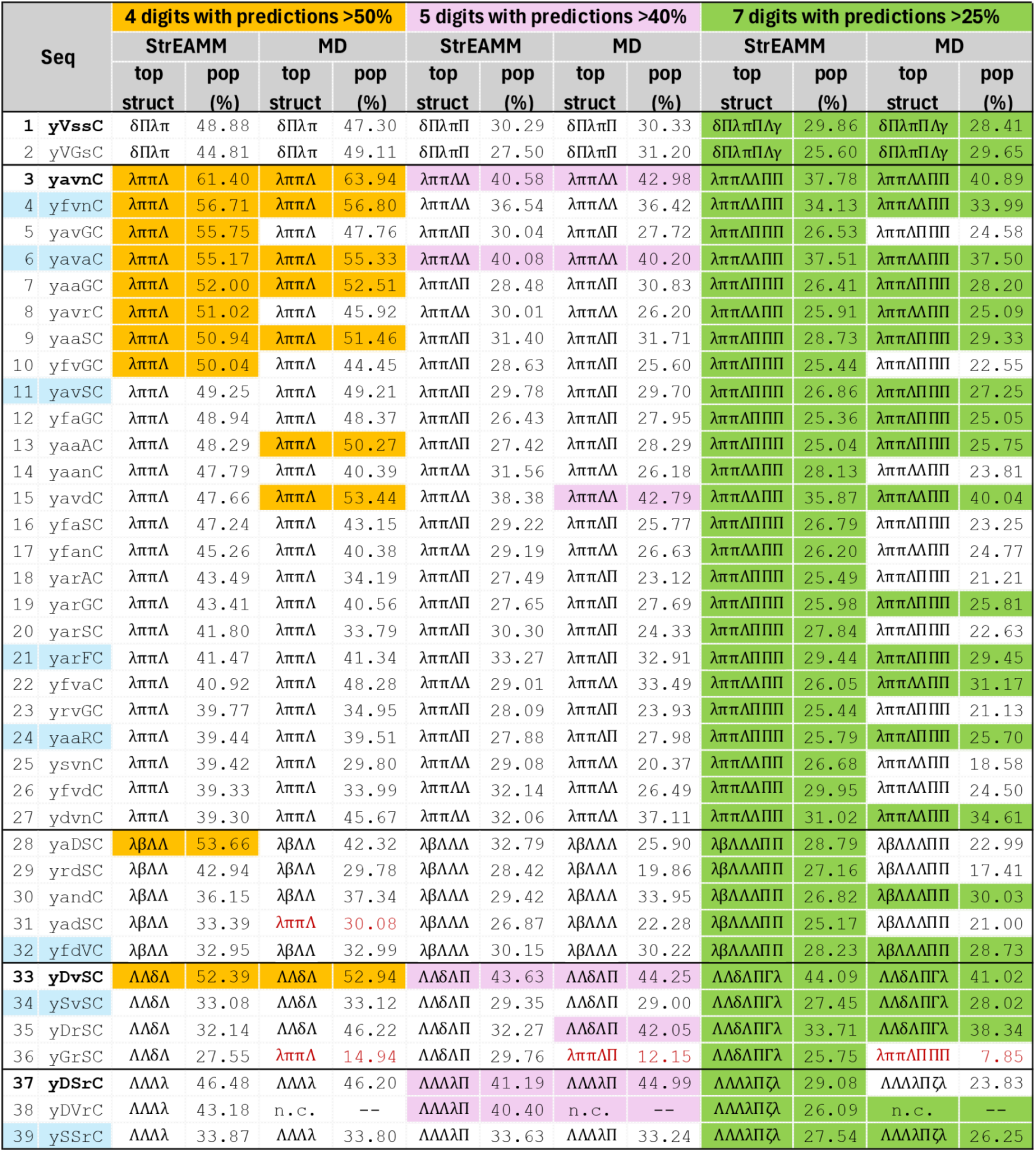
39 Best-Structured Sequences Predicted by the GNN (1,2) StrEAMM-Thioether Models^a^

a Structural predictions were made for the entire sequence space (3,375 sequences) using Models 1, 2, and 3 trained on the complete set of 396 training sequences with the optimized hyperparameters. Identified sequences in this table have a predicted top structure of at least >50% population for Model 1 (highlighted in orange), >40% population for Model 2 (highlighted in pink), or >25% population for Model 3 (highlighted in green). Of these 39 sequences, 8 are from the training data set and are highlighted in blue. The top structural digit strings from MD simulations and their corresponding populations were shown, and they were colored in red when the structural digit strings did not match those from the models’ predictions. Bolded sequences were selected for synthesis and solution NMR validations. Abbreviation: n.c. = not converged.

### Structural Analysis

Structural binning maps for each amino acid of the scaffold, as well as two additional binning maps depicting the four linker dihedral angles along the thioether linker (i.e., a map for *d*_1_–*d*_2_, and a map for *d*_3_–*d*_4_; Figures 2 and 3), were derived using our training data set by following the protocol in our previously published work on StrEAMM models^17,18^ for head-to-tail cyclic peptides (Figure 3). Briefly, the 2D dihedral distribution for each map was binned into a 100 × 100 grid, and the probability density at each grid point was computed. Subsequently, a density-peak-based method^69^ was employed for clustering using all 100 × 100 grid points of each structural binning map, resulting in 9, 9, 9, 9, 8, 9, and 6 clusters for maps d-Tyr1, R2, R3, R4, Cys5, *d*_1_–*d*_2_, and *d*_3_–*d*_4_, respectively.

### StrEAMM Neural Network Models for Thioether-Linked Cyclic Pentapeptides

We utilized GNNs in our work for their powerful applications in property predictions and structural predictions of biological and therapeutic molecules.^18,70–72^ Unlike our StrEAMM GNN models for head-to-tail cyclic peptides, which have five/six nodes for cyclic penta-/hexapeptides, respectively, here, a graph with three nodes was used to represent the three variable amino acids R2, R3, and R4 of our thioether-linked scaffold because d-Tyr and Cys are always present in our sequences (Figure 4). A 2,048-bit molecular Morgan fingerprint^73^ was used as an input node feature for each amino acid with RDKit version 2021.03.05.^74^ Besides having bidirectional edges between R2 and R3 and between R3 and R4 (Figure 4A), we also included another set of edges between R2 and R4 in another GNN model (Figure 4B). We referred to GNN models with these two different sets of edges as GNN (1,2) and GNN (1,2)+(1,3).

**Figure 4:**
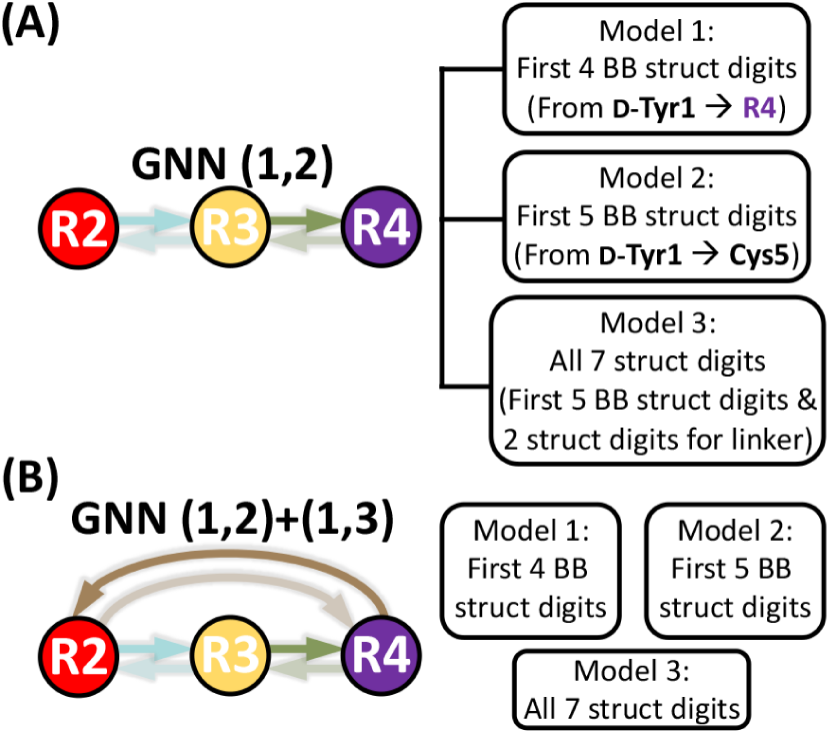
Graph neural network (GNN) architectures. (A) GNN with (1,2) edge types, denoted as GNN (1,2). Since all the thioether-linked cyclic peptides in this work share common d-Tyr1 and Cys5 residues, these two residues are not included in the graph. This architecture is used to train three separate models. Each model represents a different emphasis on the scaffold ring. Model 1 focuses on the four backbone (BB) structural digits of the first four residues. Model 2 focuses on the first five residues. Model 3 considers the first five residues and the linker’s orientation, which is represented by two additional structural digits. (B) Another GNN architecture, GNN (1,2)+(1,3), is used with additional (1,3) edge types connecting the two outer nodes. Similar to the GNN (1,2) models, it is used to train three separate models using three sets of structural digits.

After feeding the fingerprint inputs to the GNN, one message-passing operation was performed to update each node’s representation using the RGCNConv^75^ operator in PyTorch Geometric.^76^ The rectified linear unit (ReLU) activation function^77^ was applied to the learned node representations. These updated node representations were then concatenated to represent an embedding of a thioether-linked cyclic peptide sequence, which was passed through a hidden layer of a multilayer perceptron (MLP). Another ReLU activation function was applied at the end of the hidden layer. The SoftMax activation function was applied to the output of this MLP architecture to normalize the output structural ensemble vector for each sequence.

The hyperparameters of the GNN models, such as the learning rate and the number of hidden nodes in the hidden layer, were tuned using a grid search with 3-fold cross-validation (i.e., each model was trained on two-thirds of the training data set and validated on the remaining one-third) for up to 1,400 epochs and saving the model results every 100 epochs. The optimal number of epochs was selected using an overfitting criterion^78^ based on percentage change and generalized error calculation using the average *R* ^2^ at every 100 epochs over the three cross-validation data sets (Figure S2). The optimal hyperparameters (Figure S3) were used to train the final model using the complete training data set (Figure 6, “396 training sequences”) and were evaluated on the test data set (Figure 6, “50 test sequences”).

The Adam optimizer,^79^ the summation of the squared error loss function, PyTorch 2.3.0,^80^ and PyTorch Geometric 2.6.1 ^76^ were used for model training.

### Peptide Synthesis and Purification

Peptides were synthesized by Fmoc solid-phase synthesis on NovaPEG Rink Amide resin (loading: 0.47 mmol/g) using a Syro Wave automated peptide synthesizer (Biotage), as previously described.^81^ The crude peptides were subsequently chloroacetylated N-terminally with ClAc-NHC. After cleavage, peptides were cyclized in solution by treatment with triethylamine (TEA) in DMSO for 1 h under rotation, forming a thioether bond between the N-terminal chloroacetamide and the Cys thiol group. Cyclized peptides were purified by reverse-phase HPLC (Shimadzu Prominence) on a C18 column (21.2 × 250 mm, Agilent) using a linear gradient of 5–30% acetonitrile/0.05% trifluoroacetic acid (TFA). Peptide identity was confirmed by Select Series Cyclic Ion Mobility-Mass Spectrometer (Waters), and purity was assessed by ultraperformance liquid chromatography (UPLC). Fractions containing the desired product (>95% purity) were lyophilized and stored at −20 °C until use.

### NMR Characterization

Lyophilized peptides were dissolved in 90:10 H_2_O:D_2_O at concentrations of approximately 2 mM, and the pH was adjusted to be 4.8 ± 0.1. 1D and 2D ^1^H NMR spectra were recorded at 20 °C on a Bruker 600 MHz spectrometer. Standard pulse programs from the Bruker library were used, with mixing times of 60 ms for the TOCSY and 200 ms for the ROESY. Chemical shifts were referenced to sodium trimethylsilylpropanesulfonate (DSS) at 0 ppm. The peptides had well-dispersed ^1^H NMR spectra, and ^1^H, ^13^C, and ^15^N chemical shifts were assigned using TOCSY, ROESY, and ^1^H–^13^C and ^1^H–^15^N HSQC spectra and the CCPNMR Program (Tables S2–S5). Distance restraints were derived from the experimental rotating-frame nuclear Overhauser effect (NOE) data (Tables S6–S9).^82^

### Simulated Annealing

The NMR-derived structures of the four selected thioether-linked cyclic peptides were generated from simulated annealing simulations using the distance restraints derived from the experimentally determined NOEs. For all these simulated annealing simulations, the NOE-based distance restraints were applied with a force constant of 1,000 kJ·mol^–1^·nm^–2^. For each peptide, two parallel simulations using two different initial structures, NMR1 and NMR2, were performed. Each initial structure was first energy-minimized in vacuum, and this energy-minimized structure was used for 1,000 replicas with randomly generated initial velocities at 300 K. Each replica was annealed from 300 to 800 K in vacuum for 100 ps and kept at 800 K for 5 ns in an NVT ensemble. Each obtained structure from the vacuum NVT was solvated using a pre-equilibrated box of water molecules, with the minimum distance between the thioether-linked cyclic peptides and the box walls being ≥1 nm. Simple counterions (Na^+^ or Cl^–^) were added to neutralize the charge of the system, if needed. Each solvated system was energy-minimized using the steepest descent algorithm. Then, each system was equilibrated for 500 ps at 300 K in an NVT ensemble. Lastly, each equilibrated system was subjected to an annealing process in an NPT ensemble at 1 bar. Specifically, the system was heated from 300 to 500 K in 100 ps, kept at 500 K for 100 ps, cooled to 300 K in 500 ps, kept at 300 K for 100 ps, and then cooled to 5 K in 200 ps.

For simulation steps in vacuum, all nonbonded interaction cutoffs and the neighbor list radius were set to 999 nm. The neighbor list was constructed once and was never updated. For simulation steps in explicit solvent, the cutoffs of nonbonded interactions and neighbor search were truncated at 1 nm. Long-range electrostatics beyond the cutoff were treated with the particle mesh Ewald (PME) method with a Fourier spacing of 0.12 nm and a cubic interpolation order of 4. A long-range dispersion correction for energy and pressure was applied to account for the cutoff of Lennard-Jones interactions.

The temperature was monitored using a V-rescale thermostat with a time coupling constant of 0.1 ps for both peptide and solvent. The pressure was regulated using a Berendsen barostat with a time coupling constant of 2.0 ps and an isothermal compressibility of 4.5 × 10^–5^ bar^–1^. The LINCS constraint algorithm was applied to bonds connecting to hydrogen atoms. The leapfrog algorithm was used for dynamic integration with a time step of 2 fs. After completion of the simulated annealing process, the NMR-derived ensembles for the four peptides were obtained from the final frames of the 1,000 replicas. Replicas whose final frames contained *cis* peptide bonds were discarded.

## Results and Discussion

### StrEAMM-Thioether GNN Models Perform Similarly with or without the (1,3) Edges

We previously published a StrEAMM linear regression model on predicting structural ensembles of head-to-tail cyclic pentapeptides.^17^ In this model, the values of weights show the partial free energy contribution of an amino acid pair adopting a specific structural digit pair and are thus interpretable. While this model demonstrated excellent performance for head-to-tail cyclic pentapeptides, it showed relatively poor performance for head-to-tail cyclic hexapeptides, which motivated us to employ neural networks to capture complicated, nonlinear patterns.^18^ In order to prepare labeled data for training, validation, and testing in Models 1, 2, and 3 (Figure 4), structural analysis was performed to discretize the MD structural ensembles of the data sets in three distinct corresponding versions. Then, we trained GNN (1,2) models to predict the structural ensembles of these thioether-linked cyclic pentapeptides with different emphases on the cycle. From 3-fold cross-validation, our GNN (1,2) models predicted the first four backbone structural digit strings of these cyclic peptides with *R* ^2^ = 0.852 ± 0.019, the first five backbone structural digit strings with *R* ^2^ = 0.837 ± 0.015, and all five amino acids and the thioether linker orientation with *R* ^2^ = 0.790 ± 0.015 (Figures 5, “(1,2)”, and S4, “132 validation sequences”, (1,2)). We noticed that the model performance decreases as we increase the number of structural digits and, thus, the level of structural details the models need to predict. We attributed this observation to the inherent flexibility of the thioether-linked cyclic peptides, and it can be more challenging to predict the (smaller) populations when the models are responsible for predicting all of the structural digits within the complete cycle.

**Figure 5:**
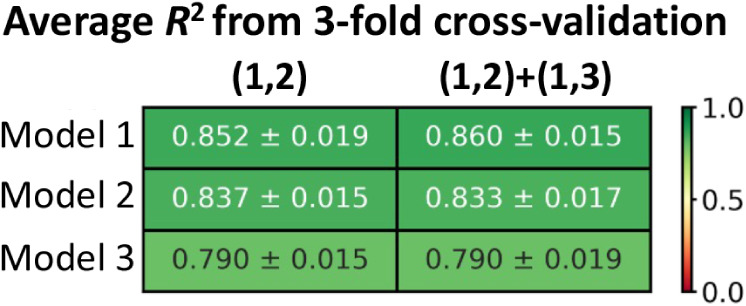
Hyperparameter tuning results from 3-fold cross-validation. Average *R* ^2^ and standard deviation values are computed using validation sets. The *p*-values between GNN (1,2) and GNN (1,2)+(1,3) for Models 1, 2, and 3 are 0.640, 0.831, and 0.981, respectively, indicating no significant difference when training models with or without (1,3) edge types.

Next, we evaluated whether the StrEAMM-thioether GNN models would perform better when considering the additional (1,3) edges (Figure 4B) to represent the potential neighbor interactions between these two outer nodes. The StrEAMM-thioether GNN (1,2) and GNN (1,2)+(1,3) of Model 1, which predict the first four backbone structural digits for d-Tyr1, R2, R3, and R4, had average *R* ^2^ = 0.852 ± 0.019 and 0.860 ± 0.015, respectively (Figure 5). Similarly, StrEAMM-thioether GNN (1,2) and GNN (1,2)+(1,3) of Models 2 and 3 had similar *R* ^2^ performances (Figures 5 and S4). Statistical tests from the cross-validation results showed no significant difference between GNN (1,2) and GNN (1,2)+(1,3), with *p*-values of 0.640, 0.831, and 0.981 for Models 1, 2, and 3, respectively. Since the neural network is capable of learning nonlinear, complex interactions, the explicit inclusion of interactions among all nodes may not be necessary, as illustrated by the similar performances of GNN (1,2) and GNN (1,2)+(1,3) across Models 1, 2, and 3.

### Use StrEAMM-Thioether GNN Models to Identify Best-Structured Sequences for Experimental Validation

After we observed that the neural networks perform similarly with or without the (1,3) edges, we focused on the StrEAMM-thioether GNN (1,2) models and trained the final models with optimal hyperparameters found from the grid search using the full training data set (Figure 6, “396 training sequences”). The performances on the test data set for Models 1, 2, and 3 report *R* ^2^ values of 0.874, 0.848, and 0.798, respectively (Figure 6, “50 test sequences”), which follow a trend similar to that observed in the hyperparameter tuning results (Figure 5).

**Figure 6:**
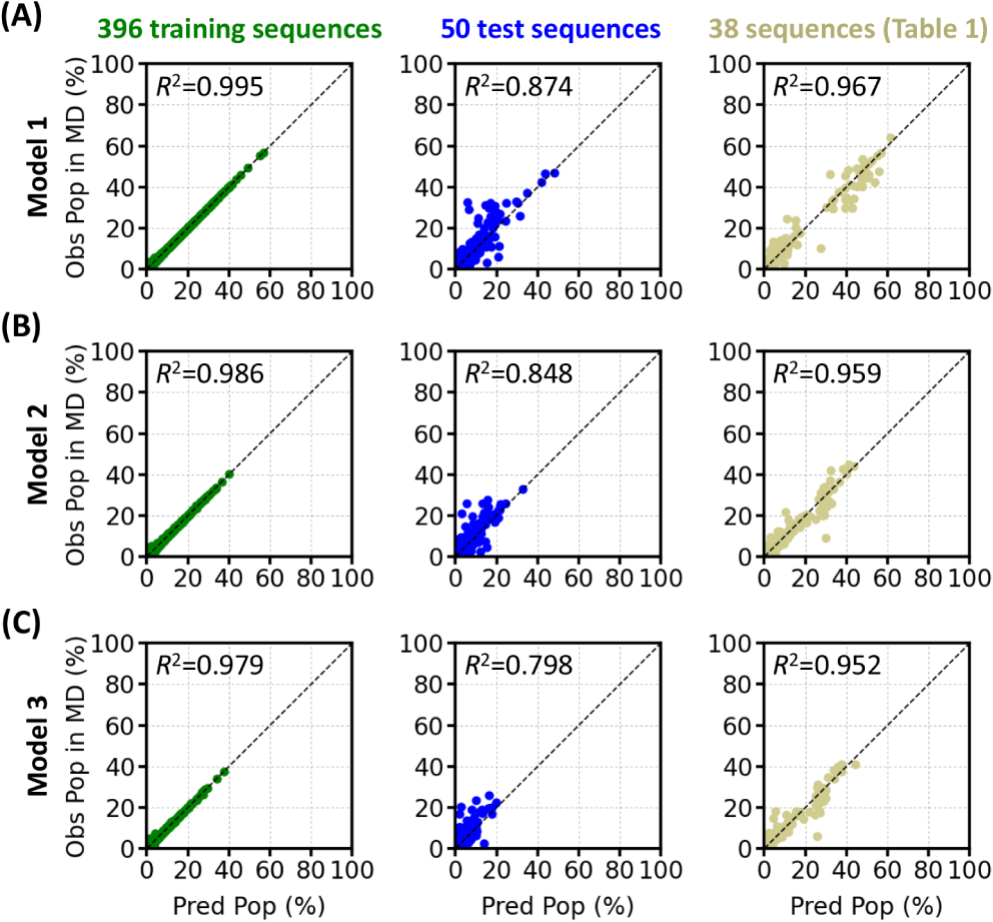
Performances on the training and test data sets, and 38 converged sequences from Table 1 using the optimal hyperparameters identified from 3-fold cross-validation for GNN (1,2). Panels A-C show *R* ^2^ values across the three data sets for Models 1, 2, and 3, respectively.

We then used these models to make predictions of the entire sequence space (15^3^ sequences) and identify the best-structured sequences. By combining structural predictions and corresponding population percentages above 50%, 40%, and 25% from Models 1, 2, and 3, respectively, this search returns 39 sequences deemed to be best-structured (Table 1). Of these 39 sequences, 8 are from the training data set, and the remaining 31 do not overlap with either the training or test data sets. Generally, the structural digit strings and populations predicted by the StrEAMM-thioether models are in good agreement with the MD results, especially the top structure (Figure 6, “38 sequences (Table 1)”, and Table 1). However, there are indeed a few inconsistencies. For example, while Model 1 predicts the top structure to be “λβΛΛ” with a population of 33.39% for Sequence No. 31, yadSC, MD simulations show that the top structure is “λππΛ” with a population of 30.08%. Upon checking the top three structures in MD for this sequence, structure λβΛΛ is the second best, with 29.42%, which is only 0.66% lower than the top structure. Similarly, although the StrEAMM-predicted top structures do not agree with the MD results for Sequence No. 36, yGrSC, the predicted top structures (ΛΛδΛ, ΛΛδΛΠ, ΛΛδΛΠΓλ) have the second-highest populations (10.02%, 9.28%, and 6.11%) in MD.

Because the pentapeptides considered here are cyclized via an N-terminus to side-chain thioether bond, their ring size (18 atoms) can be comparable to that of head-to-tail cyclic hexapeptides. When considering the dihedral angles forming the ring of this thioether scaffold, there are 13 torsions (excluding ω angles). Meanwhile, a head-to-tail cyclic hexapeptide and heptapeptide have 12 and 14 torsions (excluding ω angles), respectively. In this sense, the flexibility of a thioether-linked cyclic pentapeptide can range between a 6 mer and a 7 mer head-to-tail cyclic peptide. Compared to head-to-tail cyclic hexapeptides, we expected that this thioether scaffold to be generally less well-structured due to the flexibility of the thioether linker. Indeed, while well-structured head-to-tail cyclized hexapeptides with a population of top structures >50% have been previously reported,^11,18,83,84^ when considering all seven structural digits (i.e., Model 3), even the best-structured thioether-linked cyclic peptide has a population of only about 40% (Sequence No. 33, Table 1).

We also noticed in Table 1 that the majority of amino acids in the R2 and R3 positions adopt structural digits with matching chirality patterns (e.g., sequences 3 to 27 have d-amino acids at R2 and R3, and they adopt lowercase (positive φ) structural digits). Thus, we speculated whether a simple 1:1 translation from amino acid type to structural digits could be established. We surveyed the structural predictions for the top structures of the entire set of 3,375 sequences to see how often the amino acid chirality patterns match with the structural digit chirality patterns. Overall, l-amino acids tend to adopt l-structural digits (i.e., uppercase structural digits), and vice versa (Tables S10–S12). However, the structural digits adopted by a particular amino acid can vary. For instance, at the R2 position, d-Ala has a higher fraction for the structural digit β (Table S10), but most sequences in Table 1 containing d-Ala at R2 have structural digits of π. Another example, at the R3 position, d-Asp has a higher fraction for the structural digit π than for Λ (Table S11), but most sequences with d-Asp at R3 in Table 1 adopt the structural digit Λ. These best-structured sequences predicted by the GNN (1,2) models form five structural groups: δΠλπΠΛγ, λππΛΛΠΠ, λβΛΛΛΠΠ, ΛΛδΛΠΓλ, and ΛΛΛλΠζλ for Model 3 (Table 1). We selected four peptides from four different structural groups: Sequences 1 (yVssC), 3 (yavnC), 33 (yDvSC), and 37 (yDSrC), for synthesis and NMR structural verification. We did not select No. 28 from the λβΛΛΛΠΠ structural group because its MD populations were less consistent and smaller than the StrEAMM predictions.

Representative 3D structures of the top structures of these four peptides are shown in Figure 7. The top structural clusters of peptides 1 and 2 (yVssC and yavnC, respectively) adopt β-turn-like conformations (Figure 7A and B). More specifically, in the top structure of peptide 1, Val2 and d-Ser3 form a type II β turn,^16^ and the C=O of d-Tyr1 and the N–H of d-Ser4 form an intramolecular hydrogen bond. In the top structure of peptide 2, d-Val3 and d-Asn4 form a type II’ β turn.^16^ On the other hand, the top structures of peptides 3 and 4 (yDvSC and yDSrC, respectively) adopt more loop-like conformations compared to those of peptides 1 and 2 (Figure 7C and D). However, we note that these four cyclic peptides are still fairly flexible but should have the best chance of yielding relatively clean, interpretable NMR spectra.

**Figure 7:**
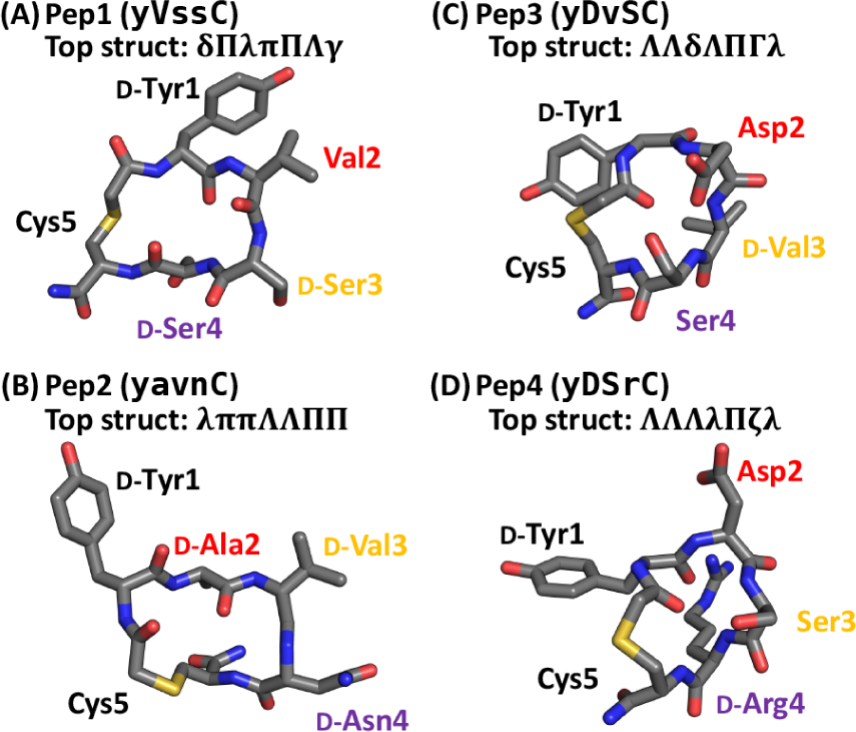
Example of the 3D conformation for the top structure of each selected peptide. (A) Peptide 1 (yVssC). (B) Peptide 2 (yavnC). (C) Peptide 3 (yDvSC). (D) Peptide 4 (yDSrC).

### NMR-Derived Structural Comparison of Four Selected Thioether-Linked Cyclic Peptides

The four peptides, yVssC, yavnC, yDvSC, and yDSrC, predicted to be best-structured by the final GNN (1,2) models (Figures 6 and 7) were synthesized, purified, and then characterized with solution NMR. Using the NOE-based distance restraints, we performed simulated annealing to derive the NMR ensembles for these selected molecules and then compared the results with MD ensembles. The dihedral distributions per residue and along the thioether linker of the NMR-derived ensembles are in agreement with the MD results (Figures S5–S8). As shown by the NMR and MD results, these peptides are flexible and do not have a single representative structure (Table 1 and Figures S5–S8).

While RMSD and radius of gyration are commonly used to distinguish conformational states,^85–89^ we did not use these metrics to quantify simulation convergence in our cases. This is because, for example, two head-to-tail cyclic pentapeptide conformations can have a seemingly small RMSD (<1 Å) but have very different (φ, ψ) values for one or more residues. To further assess the conformational ensembles between MD and NMR results, we performed dPCA^67^ and computed NIP^68^ between MD and NMR ensembles for each peptide in the space spanned by the top three principal components to assess overlap between the ensembles (see “Molecular Dynamics Simulations and Principal Component Analysis” in Materials and Methods). Good convergence was observed between MD1 and MD2 and between NMR1 and NMR2 (Figure 8A and B). We observed poorer overlaps and lower NIPs between the MD and NMR results (Figure 8, see comparison results for peptides 2–4 in Figure S9). This is expected because simulated annealing is not an enhanced sampling approach aiming to report the equilibrium free energy profile and provide a converged structural ensemble; it uses high temperatures and cools the system to discover structures that satisfy NMR-based restraints. Thus, potential structural ensemble discrepancies are likely to be inevitable for flexible cyclic peptides.

**Figure 8:**
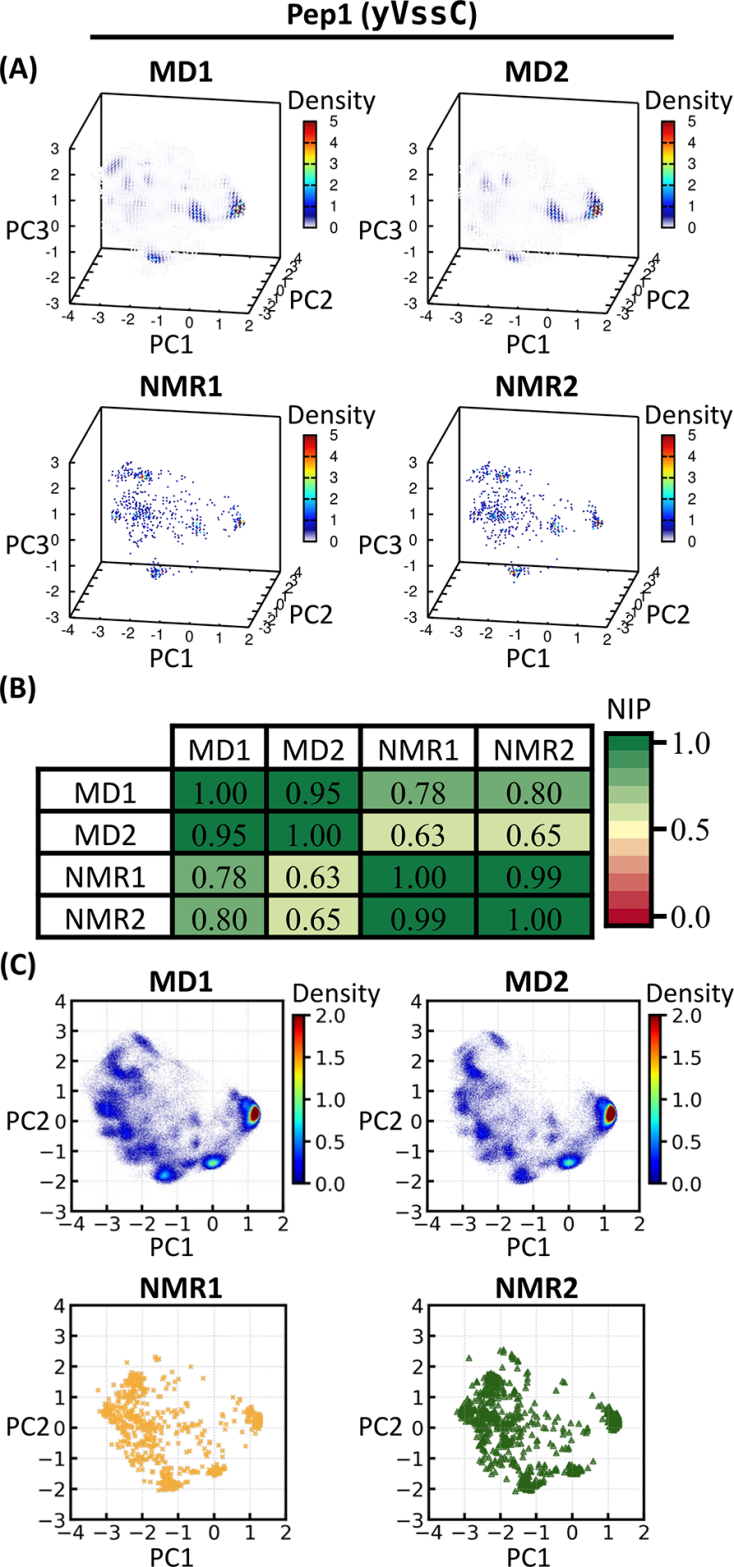
dPCA projection and NIP calculation for MD1, MD2, NMR1, and NMR2 of peptide 1 (yVssC) from two independent BE-META and simulated annealing runs, respectively. (A) The projection of all trajectories onto a common 3D PC space using the first three PCs. (B) NIP values for the densities of all MD and simulated annealing runs. A Gaussian kernel with σ = 0.5 was used for each data point in the 3D PC space. (C) The projection of all trajectories onto a common 2D PC space like panel (A) for easier visualization and comparison of the MD and NMR-derived ensembles.

We further analyzed the seven structural digits of the simulated annealing results to calculate the populations for different structural strings and then compared their top five structural clusters with those observed in MD (Table 2). We observed that most of these top structural digit strings seen in MD are often seen in the simulated annealing results. Figures S10–S13 further show the NOE violations for each of the top five structural digit strings obtained from MD and simulated annealing for the four peptides. Generally, the top structural digit strings seen in MD have a similar number of violations as those in the simulated annealing results. The violation statistics from all the frames are provided in Tables S6–S9, complementing Figures S10–S13. These experimental NOEs represent time- and ensemble-averaged contacts. When a cyclic peptide is flexible and samples multiple conformations, it is likely that no single conformation can satisfy all distance restraints simultaneously. Forcing all violations to zero would require overconstraining the calculations and would produce distorted nonphysical conformations. We also note that many of the NOE-based distance restraints observed for peptides 1–4 have long upper bounds, which provide little utility for defining a single conformation. However, these restraints also have lower bounds, which can still contribute to characterizing the conformational ensemble. Our goal is not necessarily to determine a single representative structure but rather to represent the conformational ensemble, which can offer a more accurate depiction of these flexible peptides’ behavior in water. Taking this and the use of simulated annealing into account, we consider that the MD and NMR results are in general agreement.

**Table 2:**
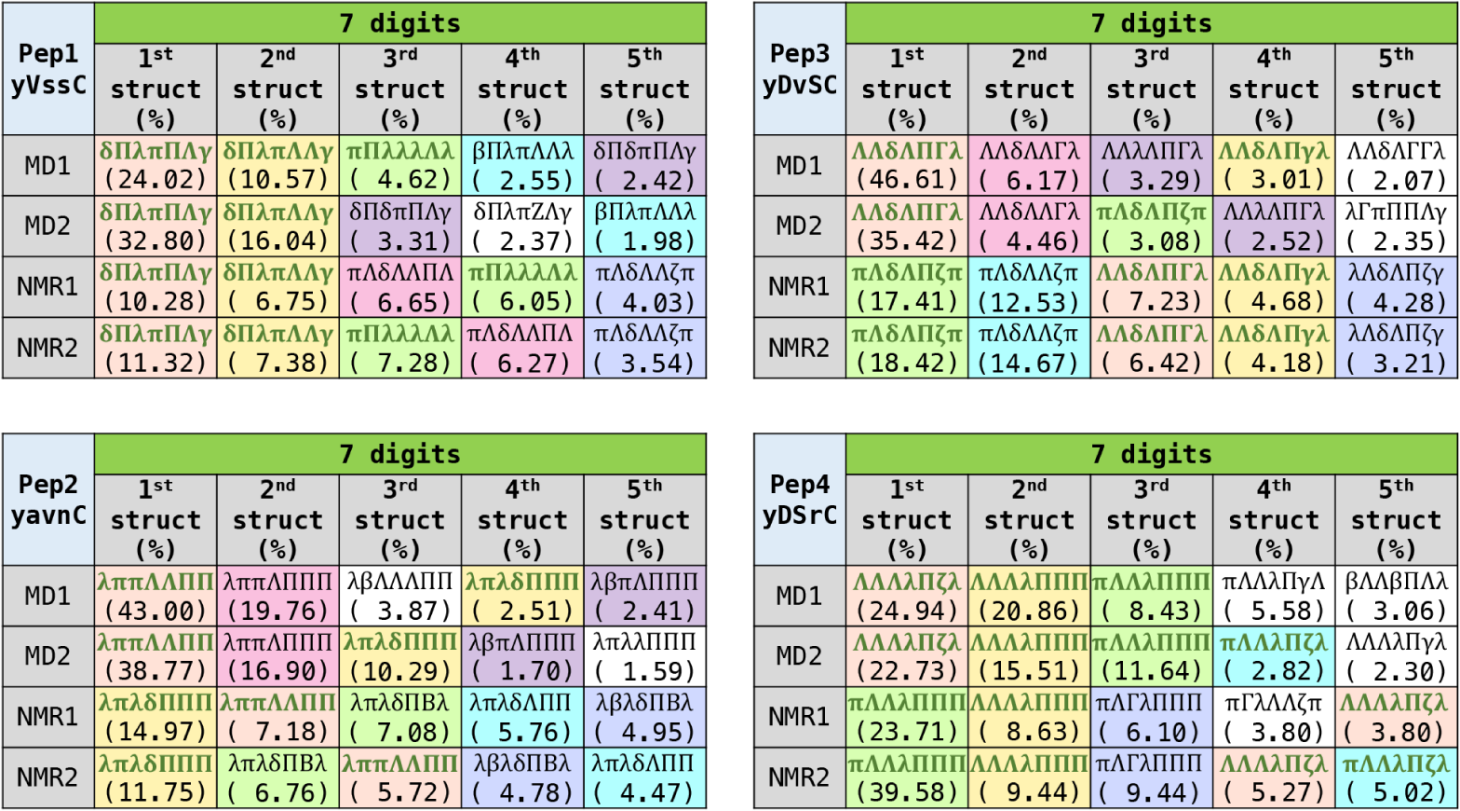
Comparison of the Top Five Clusters from MD- and NMR-Derived Ensembles of the Four Selected Thioether-Linked Cyclic Peptides: yVssC, yavnC, yDvSC, and yDSrC^a^

a The structural digit strings that appear in both MD- and NMR-derived structures are in dark green. The population of each structural digit string is shown in parentheses. The cells in each row are colored when a structural digit string overlaps.

## Conclusions

Here, we report the application of the StrEAMM method to predict structural ensembles of thioether-linked cyclic pentapeptides. Toward this goal, we perform BE-META simulations on training sequences to obtain the structural digit strings adopted by the cyclic peptides and their corresponding populations. We train GNNs using Morgan fingerprints to represent the amino acids in our library and labeled data from MD simulations.

Our models perform well with an average *R* ^2^ of 0.874, 0.848, and 0.798 on the test data set for Models 1, 2, and 3, respectively (Figure 6, “50 test sequences”). We acknowledge that as we increase the structural resolution of the predictions (e.g., going from the first four backbone structural digits to seven structural digits for the full cycle), the model performance decreases. This could potentially be due to the flexible nature of this class of cyclic peptides, resulting in more structures with lower populations, which are likely more difficult to predict accurately when using all the digits to describe the structure of the full cycle. Additionally, as more structural regions appear in these binning maps (Figure 3) than in those for head-to-tail cyclic penta-/hexapeptides,^17,18^ this results in different structural digit string clusters with smaller populations, which can be challenging for the models.

The current models identify four thioether-linked cyclic pentapeptides predicted to be the best-structured. These four peptides are synthesized and characterized with solution NMR. The NOE-based distance restraints enable us to derive the NMR ensembles for these peptides, which are similar to the MD results (Table 2).

We acknowledge the therapeutic potential of larger thioether-linked cyclic peptides. In such cases, conformation sampling using BE-META can be applied with additional 2D CVs along the backbone of additional amino acids. However, as the ring size increases, conformational flexibility tends to increase and MD simulation can require more time to achieve convergence. We are exploring different strategies for the effective enhanced sampling of larger cyclic peptides. In particular, a recent work by Miao et al. showed that the sampling performance of unsupervised ML methods is on par with the conventional 2D CVs when applied to simulate head-to-tail cyclic pentapeptides, but it deteriorates as the size of the cyclic peptides increases.^90^

Proline and other *N*-methylated amino acids also serve as building blocks in cyclic peptide designs.^91–93^ However, the inclusion of such amino acids can increase the propensity for a *cis* peptide bond. The *cis*/*trans* population of peptide bonds in cyclic peptides containing these types of residues can be obtained using BE-META simulation by introducing an additional CV that bias the ω dihedral angle between each proline (or *N*-methylated amino acid) and the preceding residue. The StrEAMM method can also be adjusted to include the peptide bond configurations during both training and prediction. However, the current peptide force fields are generally not parametrized to predict the peptide bond configuration correctly, and we are working to address this issue as well.

Overall, the promising results shown herein demonstrate the application of ML capabilities to expand structural predictions of another class of cyclic peptides beyond our current models for head-to-tail cyclized peptides. We are also exploring other ML architectures, such as diffusion models, to enable the prediction of different cyclic peptide scaffolds within a single model architecture. We envision that the application of these StrEAMM models can provide structural details that can be beneficial for the rational design and development of cyclic peptides.

## Supplementary Material



## Data Availability

Tufts University will share the StrEAMM models and data used for the StrEAMM models with academic and nonprofit users under a limited-use transfer agreement. Interested commercial users can contact the Office for Technology Transfer and Industry Collaboration at Tufts University. Due to the large size of the data files, raw simulation trajectories will be made available upon request. The NMR-derived ensembles for peptides 1–4 identified from this study can be found in the public GitHub repository, https://github.com/ysl-lab/CPThioetherNMR_mho.

## Notes

Lists of sequences in the training and test data sets; chemical structure of tethered d-Tyr–Cys used for partial charges derivation of Cys with a thioether bond and the carboxyl group linking to the NH of d-Tyr; examples of learning curves from hyperparameter tuning; optimal hyperparameters used in final model training; examples of models’ performances from one of the three folds in 3-fold cross-validations; comparison between MD ensemble and NMR-derived ensemble for selected peptides; comparison of simulation convergence between MD ensemble and NMR-derived ensemble for selected peptides; violation analysis for the top five structures in MD and NMR results for selected peptides; number of converged sequences and sequences failed to converge during BE-META simulations; chemical shifts for selected peptides; NOE-based distance restraints for selected peptides and violation statistics; comparison of amino acid chirality patterns versus structural digit chirality patterns for predicted top structures of the entire sequence space at R2, R3, and R4 positions (PDF)
