# Supplementary material for: StrEAMM-Thioether: Efficient Structure Prediction for Thioether-Linked Cyclic Peptides
Source: J Phys Chem B. 2026 Feb 6;130(7):2031–43. doi: 10.1021/acs.jpcb.5c06368 (PMC13262343; doi:10.1021/acs.jpcb.5c06368)
Supplement: Supplementary file 1 [file jp5c06368_si_001.pdf]

Supplementary Information

StrEAMM-Thioether: Efficient Structure Prediction for Thioether-Linked Cyclic Peptides

Minh Ngoc Ho[1], Jiayuan Miao[1], Yi Shan[2], Choi Yi Li[3], Hiroaki Suga[3], James D. Baleja[2], and Yu-Shan Lin[1]*

[1]Department of Chemistry, School of Arts and Sciences, Tufts University, Medford, Massachusetts 02155, United States

[2]Department of Developmental, Molecular, & Chemical Biology, Pharmacology and Drug Development Program, Graduate School of Biomedical Sciences, School of Medicine, Tufts University, Boston, Massachusetts 02111, United States

[3]Department of Chemistry, Graduate School of Science, The University of Tokyo, Tokyo 113-0033, Japan

*To whom correspondence may be addressed. Email: yu-shan.lin@tufts.edu

Keywords: Thioether-linked cyclic peptides, molecular dynamics simulation, machine learning, structure prediction

List S1. 396 sequences in the training data set. This data set contains (A) 15 random sequences with each of the three variable positions (R2, R3, and R4) having all amino acids from the 15-aa library; (B) 43 sequences with each amino acid from the 15-aa library at position R2, R3, or R4, while the residues at the other two variable positions are Gly; (C) remaining 338 random sequences with no duplication.

(A)

yGndC yARGC yaSrC yVDnC yvfsC yFaaC yfvRC yNAFC ynGfC ySNvC ysVSC yDFDC ydsVC yRdAC yrrNC

(B)

yGGGC yAGGC yGAGC yGGAC yaGGC yGaGC yGGaC yVGGC yGVGC yGGVC yvGGC yGvGC yGGvC yFGGC yGFGC
yGGFC yfGGC yGfGC yGGfC yNGGC yGNGC yGGNC ynGGC yGnGC yGGnC ySGGC yGSGC yGGSC ysGGC yGsGC
yGGsC yDGGC yGDGC yGGDC ydGGC yGdGC yGGdC yRGGC yGRGC yGGRC yrGGC yGrGC yGGrC

(C)

ySNRC yGfdC yrfdC yGrNC yrfNC yGrdC yrrdC yGfNC yGavC ynSDC yVrRC yDafC yfvnC ySfVC ydNdC
yvVfC yvaSC yvGsC ynRaC yrsFC ynGSC yGSNC yDGSC yddRC yRVaC ySSVC yGAnC yaDAC ysnfC yAdGC
yNrGC yAnFC yrnVC yfDvC ynVAC yNFNC yNFVC yNVAC yfGdC yarrC yafFC ysnGC yVvsC ydDrC yVaRC
ynSrC yNGdC yddFC yVafC yFraC ynvfC ysrVC yfAFC yVAVC yANFC yANAC yGARC yrDDC yrVSC yGADC
yrNfC yFRfC ySRGC yGSVC yRVRC ysVvC yaVAC ysssC yrFNC yfAaC ysnVC yVAaC yFGsC yAsaC yFsAC
yGFDC ydNnC yvrnC yRDfC ydrFC yRvDC yrDrC yfGAC yasrC yFGfC yNsNC yRsvC yDRDC yNNVC ySnDC
yVNdC yAVfC yaFSC yAvSC yarFC ysvsC yDfaC yGaFC yrRFC yNffC yavSC ySvVC yrGdC yVdrC yVRaC
ysNnC yVsSC yGSaC yVvfC ydnSC yaDfC ynvaC yRSvC yADvC yDVGC yndNC yrGRC ydANC yFnnC yFsRC
yfFVC yrDVC yvadC yVfaC ySfnC ysvVC ydaAC yFfNC yvvVC ySRdC yVarC ysfNC yadsC yVSsC yASFC
yFfFC yrDsC yrVdC yfnaC yfrsC yvFNC yrdDC ynRFC ySSRC yFFvC yVvRC yAfnC yVnGC ySSrC yvGNC
yrVGC yADDC ydAnC ysSGC ySVdC yfdVC yvGVC yVVaC ysfVC yFnNC ySvFC ySadC yAGdC yARvC yRffC
yDnfC yfFsC yaVNC ySNDC yfVfC ydvrC yGsaC yrrVC yaaRC yasnC yGRnC ySvSC ynFnC yRNNC yNsrC
yRnAC ydDSC yrAnC yFGdC yaAAC yFsFC ynVDC yRAAC yNFaC yNVsC yGrnC yDFdC ynFfC ydDAC yasDC
yRNRC ysRNC ydNGC ynnrC ydNFC yFnDC yGDNC yAADC yFnsC yAdNC yNdvC yfndC yASDC yfvVC ySDsC
ynRRC yaGSC yFnAC yfFNC yvfFC yrfGC yrNsC yfvsC ynaAC yfNVC ydvSC yDfsC ySssC ySdVC yNfSC
yssDC yAsVC yNrrC yfafC yavaC yNAVC yfsDC yNvAC yVFvC yfnFC yNVRC yvGDC yRdrC yfasC yGRvC
ysdaC yrRSC yRRSC yddAC ySFNC yrDaC yVfVC yfFSC yVnFC yRDFC ysfAC ydDNC yrdVC yasAC yaNnC
yaNrC yvAnC yDNfC ynrSC ySNnC yavNC ynGdC ySsfC yGNSC yrFaC yDVSC yvRNC yDGAC yFFFC yAaAC

yvSdC yNddC ydVsC yGAaC yanSC yGrvC yFfSC yaRSC ydrDC yDVDC yFaDC ySrGC yfDGC yDSnC yvVaC
yaFAC ysANC yfdDC ysNNC yAArC yfraC ySdGC ySdDC yfnVC yGSvC yvSRC yRNAC ySRaC yaFfC yRrNC
ysaDC ynFaC ynGrC yRdvC ynsaC ydnFC yGnFC yNGAC yrvaC yssvC yNDSC yFsGC ySdAC yDGsC ynDDC
yVVGC yaVFC yVSvC yvAFC yrnSC yVFDC ydDdC ysaRC yGVnC yafDC yVnaC yDsGC yNNSC yFFsC ySFfC
yAsvC yFfRC yfdfC ySfrC yArVC yAAdC yRFnC yDvGC

List S2. 50 random sequences in the test data set.

yFSVC yrvnC yAnsC yRFAC yRFfC ysaAC yAdAC yFSSC yaRDC yrGVC yFGaC yvSsC yvnfC yDGvC yADFC
yVsNC yGRAC yRdsC yVdvC yDnFC yNdaC ydfdC yVfvC ynNnC yGRsC yvNaC yDAnC ydGrC ysfsC yFaFC
ysDAC yGaNC yaSDC ydDaC ydVGC yrNaC yDvAC ySfsC yFFRC ynGNC ySRRC ysrRC yFAGC ydssC yRFdC
yrffC yrrAC yNarC yGsSC yNvnC

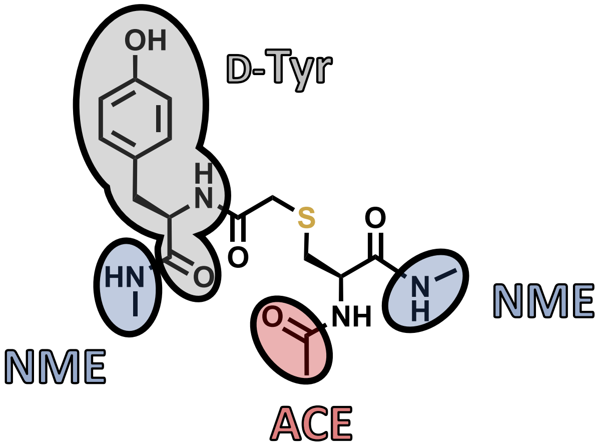

Figure S1. The chemical structure of tethered D-Tyr–Cys used for partial charge derivation of the Cys containing the thioether bond. The two *N*-methyl (NME; blue oval) caps are placed at the C-termini of both D-Tyr (gray oval) and Cys. The acetyl (ACE; red oval) cap is placed at the N-terminus of Cys. At the end of the RESP charge fitting, only partial charges of Cys with the thioether bond and the carboxyl group linking to the primary amide of D-Tyr were kept for subsequent simulations, while partial charges of ACE, NME, and D-Tyr from the charge fitting were discarded.

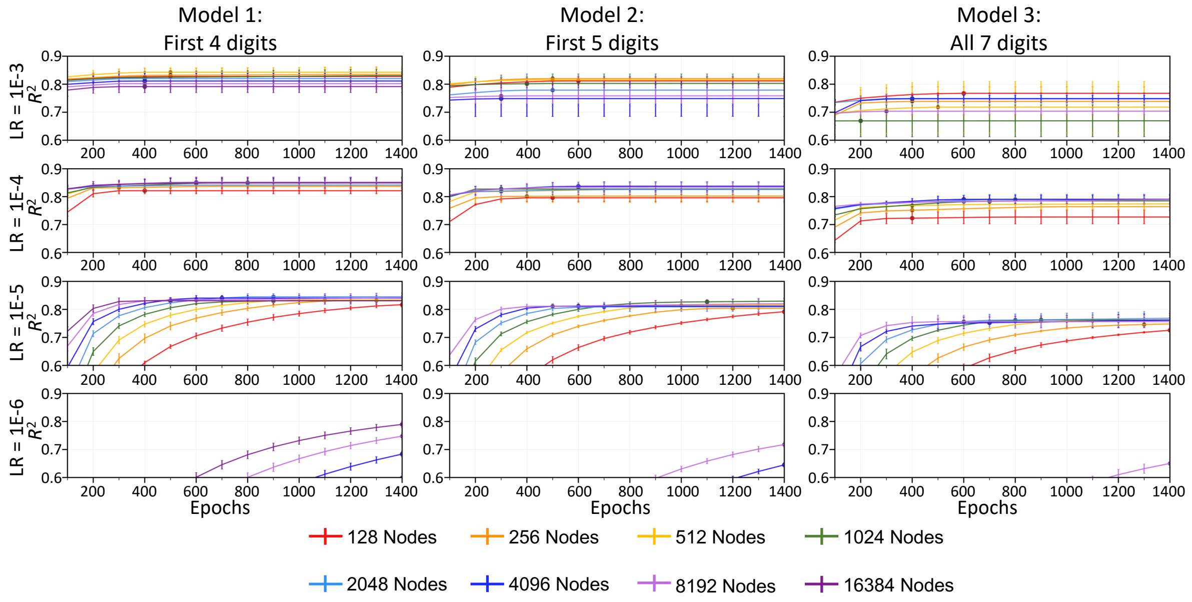

Figure S2. Example of epoch selection for the GNN (1,2) models using 3-fold cross-validation. The optimal number of epochs (indicated by filled circles) was selected based on the percentage change and the generalized error criterion. The vertical lines represent the standard deviation across the three folds from cross-validation. For example, the optimal number of epochs for Model 3, using a learning rate of 1E–3 and 1,024 hidden nodes, is 200 (top row, right column, green circle).

(A)

Optimal hyperparameters for (1,2)

	LR	Hidden nodes
Model 1	1E-4	8192
Model 2	1E-4	4096
Model 3	1E-4	4096

(B)

Optimal hyperparameters for (1,2)+(1,3)

	LR	Hidden nodes
Model 1	1E-4	2048
Model 2	1E-4	2048
Model 3	1E-4	1024

Figure S3. Optimal hyperparameters selected from the grid search. The optimal learning rate and number of hidden nodes in the hidden layer for each GNN model were selected based on the best average R^2 across the three cross-validation data sets. These selected hyperparameters were used for final model training on the complete training data set. (A) Hyperparameters selected for the GNN (1,2) architecture. (B) Hyperparameters selected for the GNN (1,2)+(1,3) architecture.

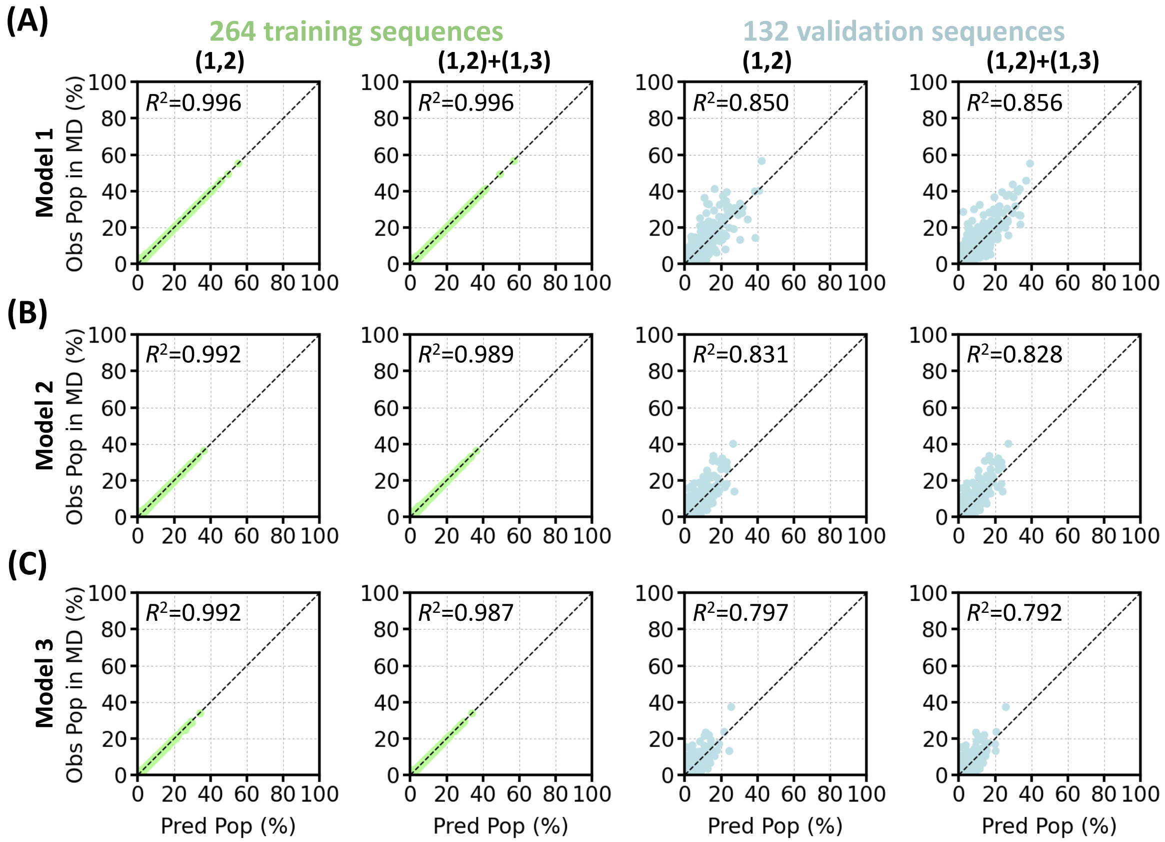

Figure S4. Examples of models' performances for one out of three folds from the 3-fold cross-validation on the training data sets, in which 2/3 were used for training and 1/3 was used for validation. Panels (A)–(C) show performances using GNN (1,2) or GNN (1,2)+(1,3) architectures for Models 1, 2, and 3, respectively.

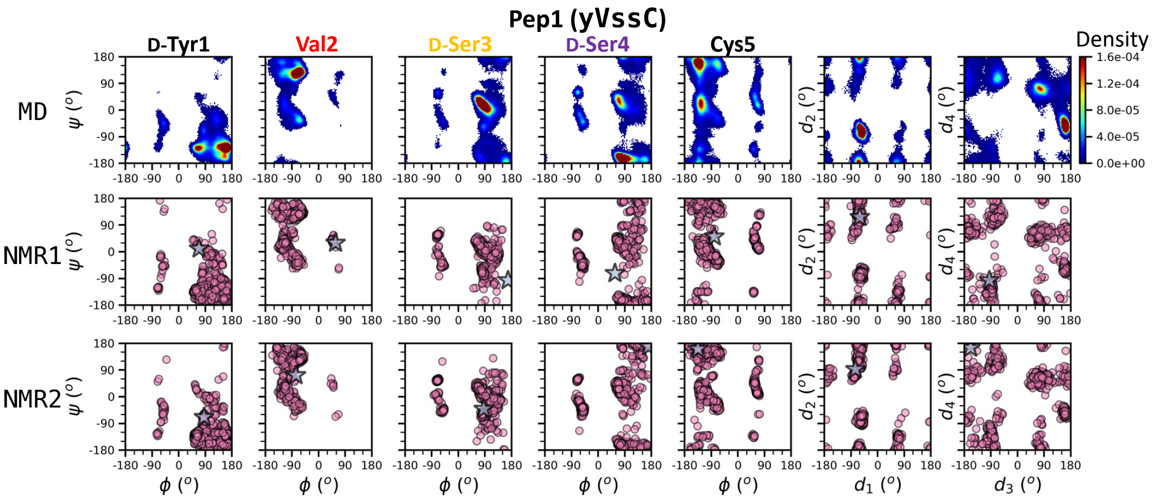

Figure S5. Comparison of the (ϕ,ψ) and linker dihedral distributions from MD (MD1 combined with MD2) and NMR-derived ensembles for peptide 1 (yVssC). The grey stars represent the dihedral angles for each amino acid and the linker in the initial structures used in two independent simulated annealing runs (NMR1 and NMR2).

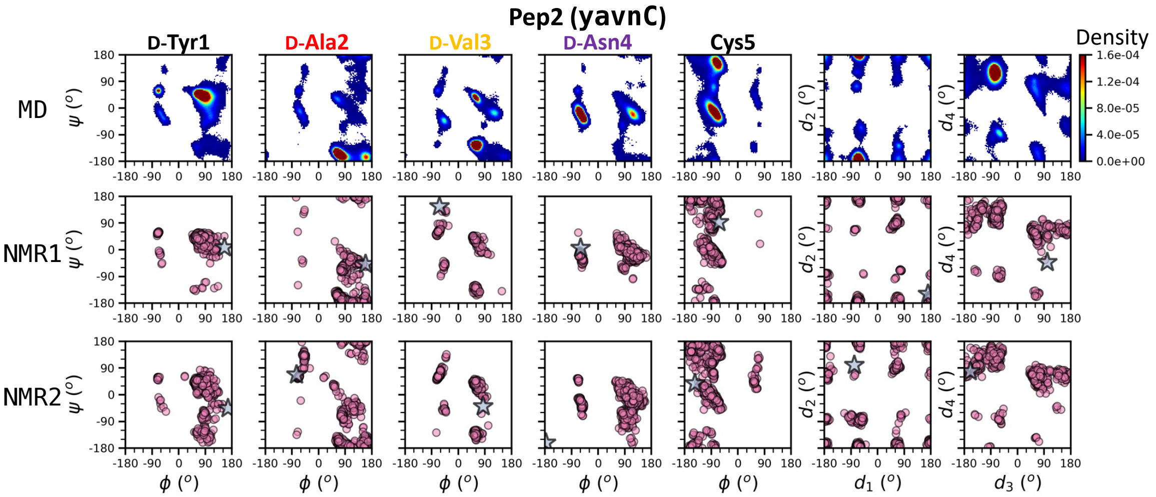

Figure S6. Comparison of the (ϕ,ψ) and linker dihedral distributions from MD (MD1 combined with MD2) and NMR-derived ensembles for peptide 2 (yavnC). The grey stars represent the dihedral angles for each amino acid and the linker in the initial structures used in two independent simulated annealing runs (NMR1 and NMR2).

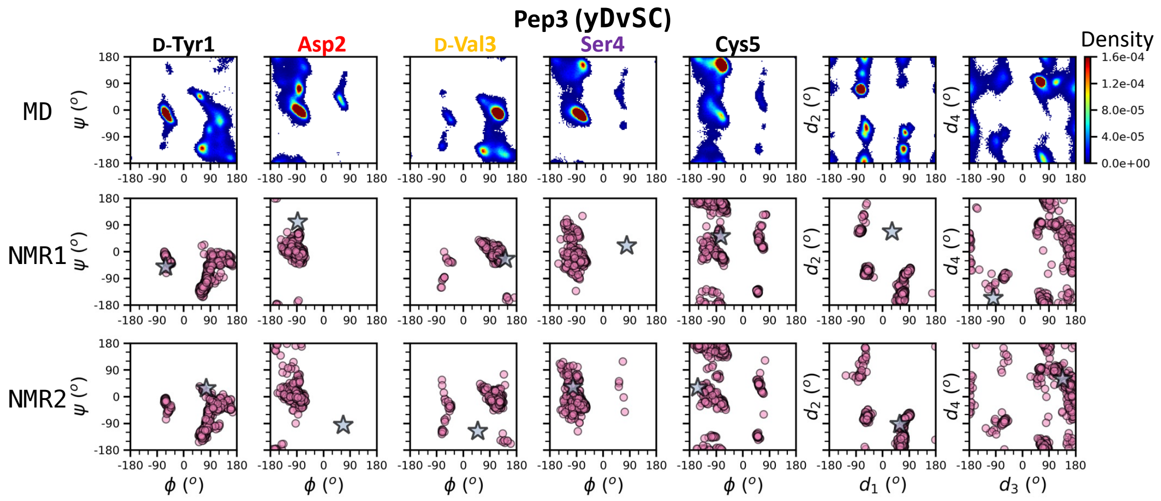

Figure S7. Comparison of the (ϕ,ψ) and linker dihedral distributions from MD (MD1 combined with MD2) and NMR-derived ensembles for peptide 3 (yDvSC). The grey stars represent the dihedral angles for each amino acid and the linker in the initial structures used in two independent simulated annealing runs (NMR1 and NMR2).

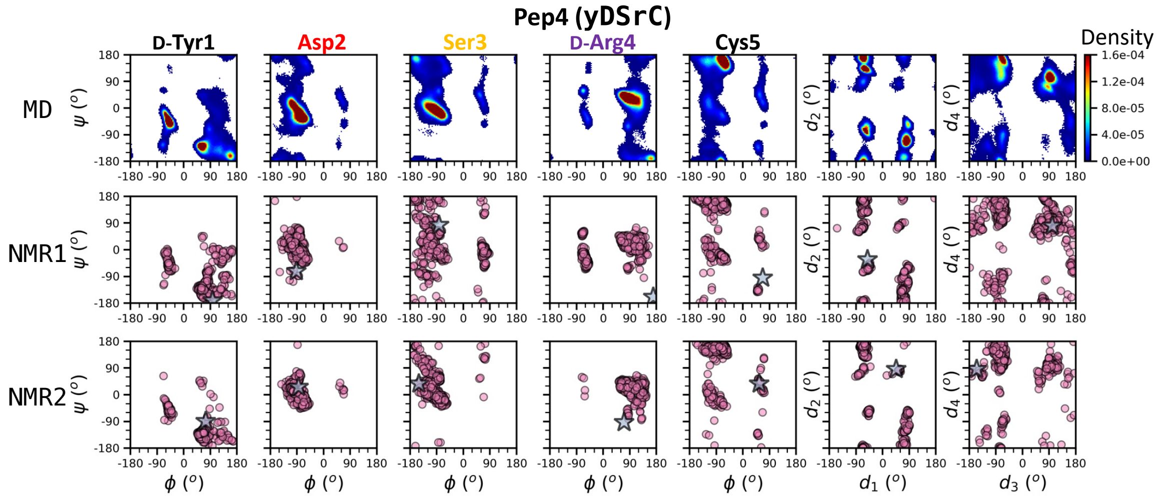

Figure S8. Comparison of the (ϕ,ψ) and linker dihedral distributions from MD (MD1 combined with MD2) and NMR-derived ensembles for peptide 4 (yDSrC). The grey stars represent the dihedral angles for each amino acid and the linker in the initial structures used in two independent simulated annealing runs (NMR1 and NMR2).

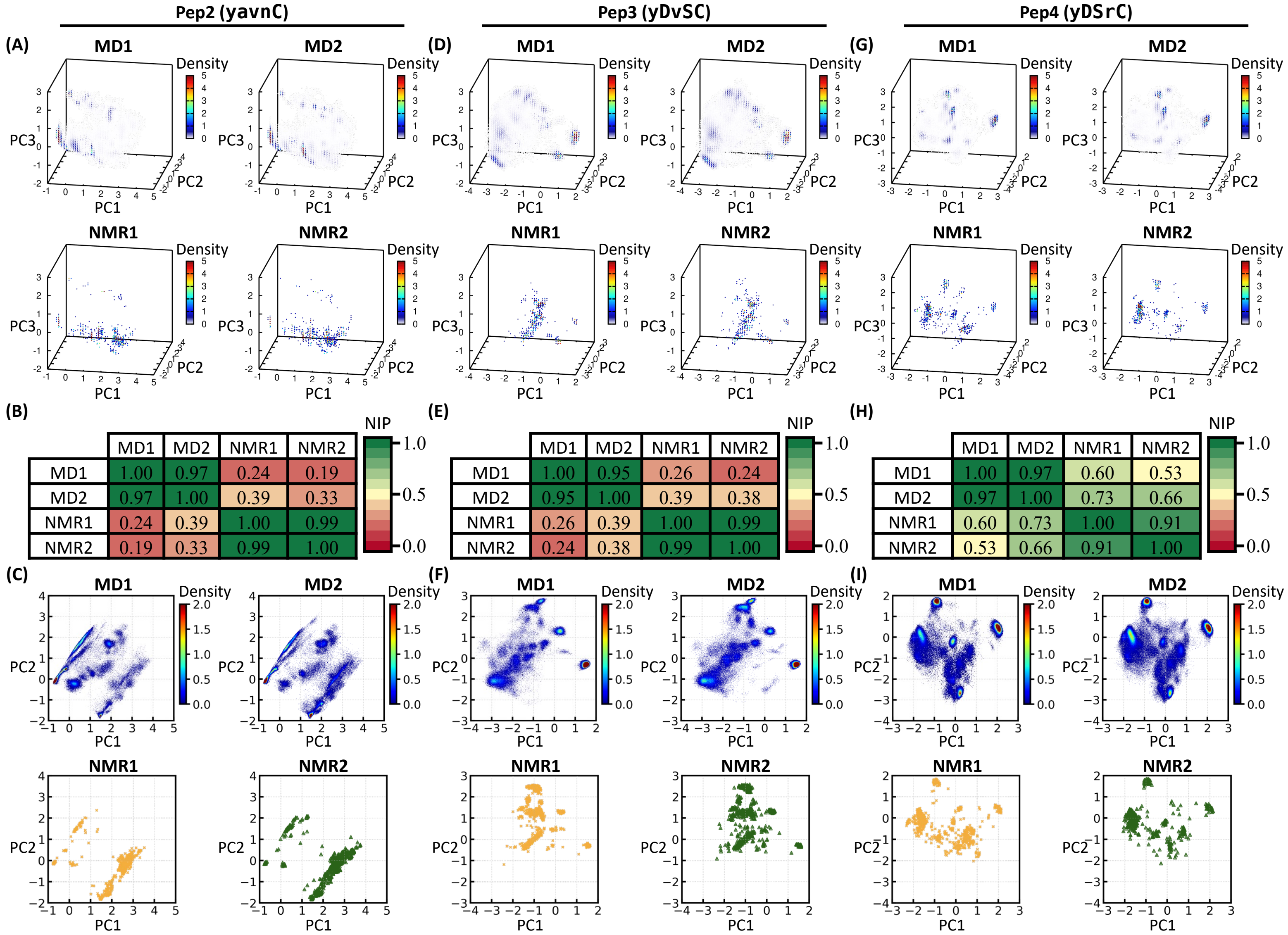

Figure S9. dPCA projection and NIP calculation for MD1, MD2, NMR1, and NMR2 from two independent BE-META and simulated annealing runs, respectively, for peptide 2 (yavnC), peptide 3 (yDvSC), and peptide 4 (yDSrC). (A) The projection of all trajectories onto a common 3D PC space using the first three PCs for peptide 2. (B) NIP values for the densities of all MD and simulated annealing runs for peptide 2. A Gaussian kernel with σ=0.5 was used for each data point in the 3D PC space. (C) The projection of all trajectories onto a common 2D PC space for peptide 2 like panel (A) for easier visualization and comparison of the MD and NMR-derived ensembles. (D–F) similar outline for peptide 3; (G–I) similar outline for peptide 4.

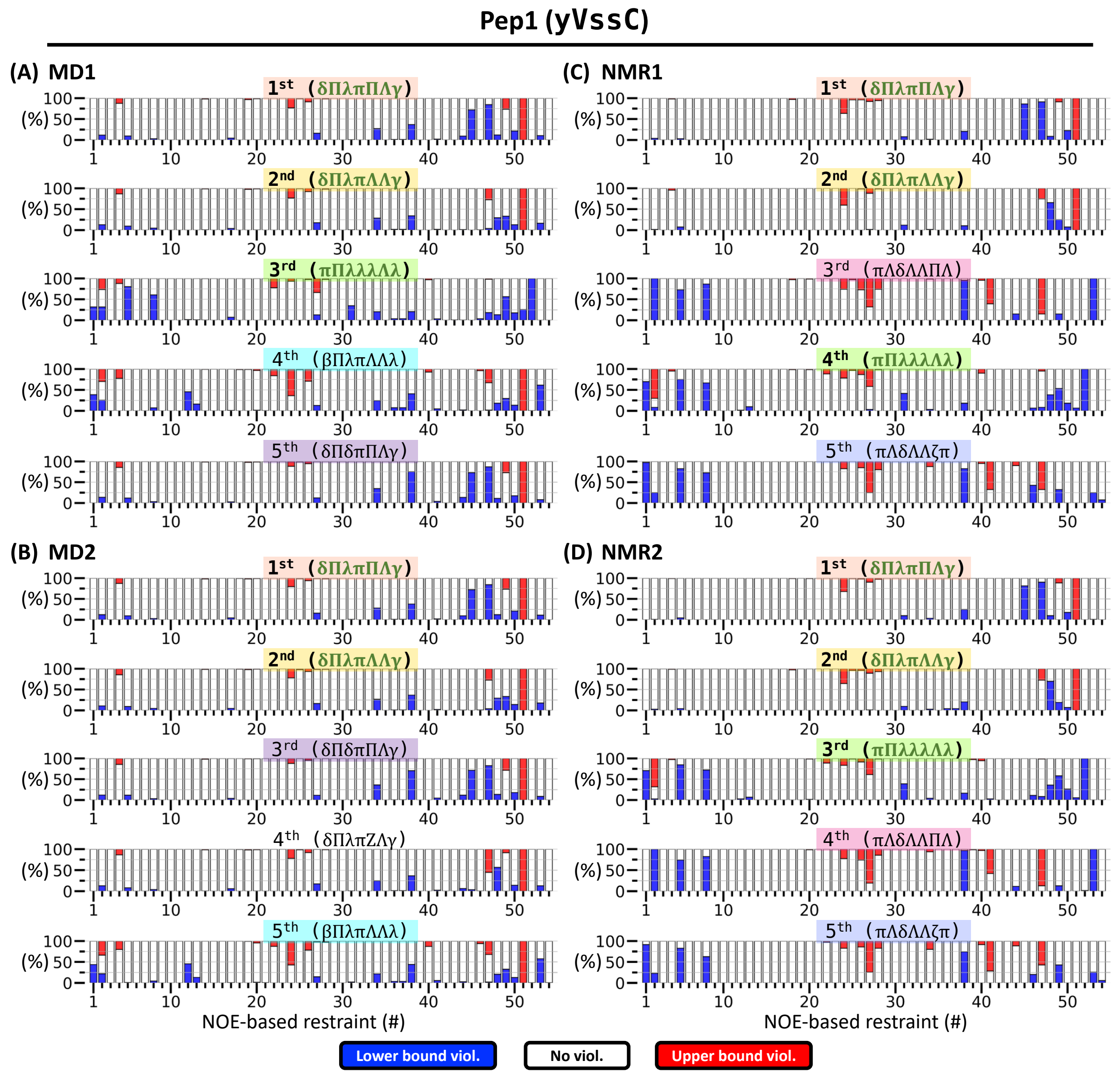

Figure S10. Violation analysis for the top five structures of peptide 1 (yVssC). The percentages of frames with lower-bound and upper-bound violations are colored in blue and red, respectively. Panels (A)–(D) show the violation patterns from MD1, MD2, NMR1, and NMR2, respectively. The structural digit strings are colored as in Table 2. See Table S6 for the NOE-based distance restraints.

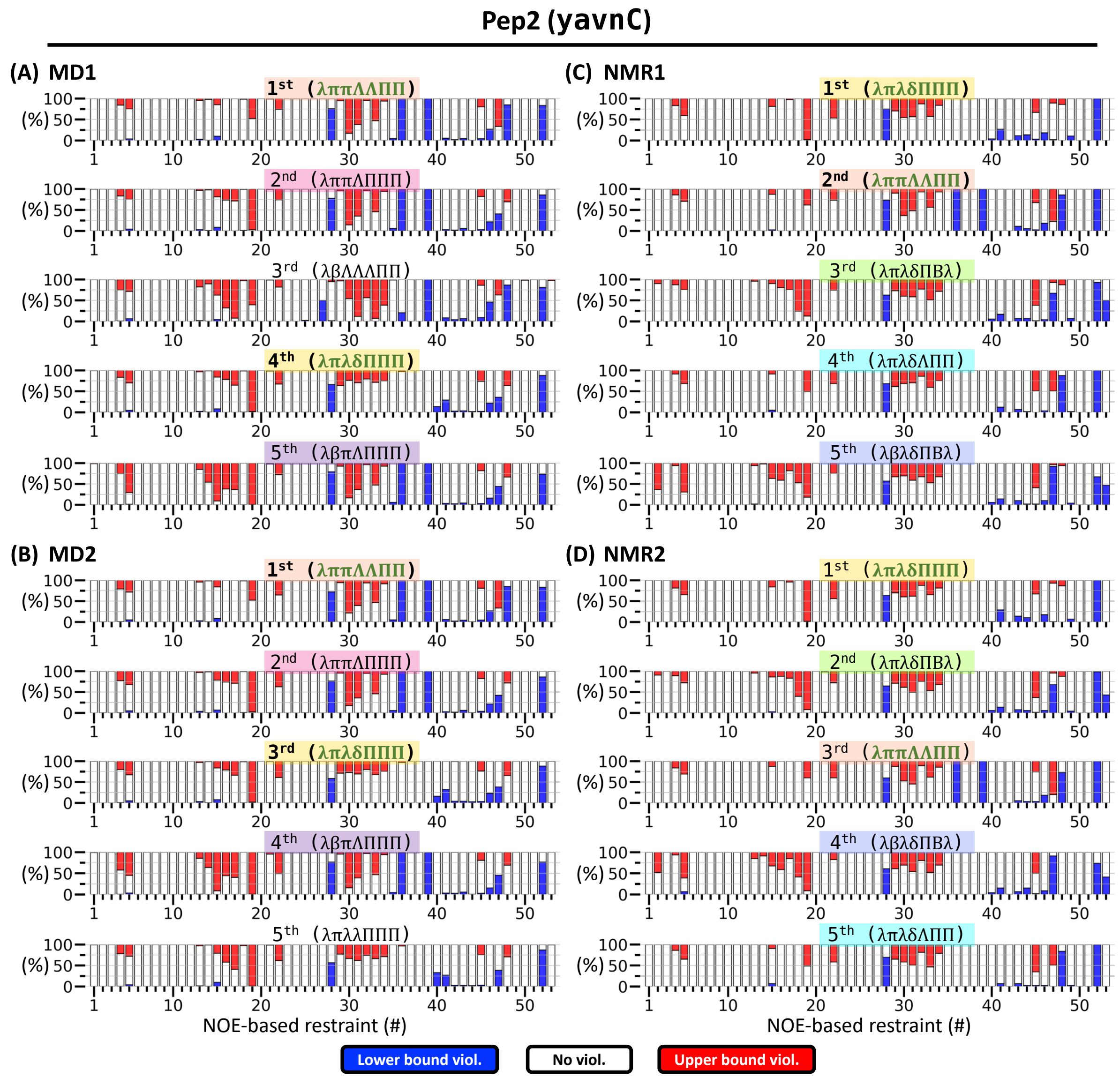

Figure S11. Violation analysis for the top five structures of peptide 2 (yavnC). The percentages of frames with lower-bound and upper-bound violations are colored in blue and red, respectively. Panels (A)–(D) show the violation patterns from MD1, MD2, NMR1, and NMR2, respectively. The structural digit strings are colored as in Table 2. See Table S7 for the NOE-based distance restraints.

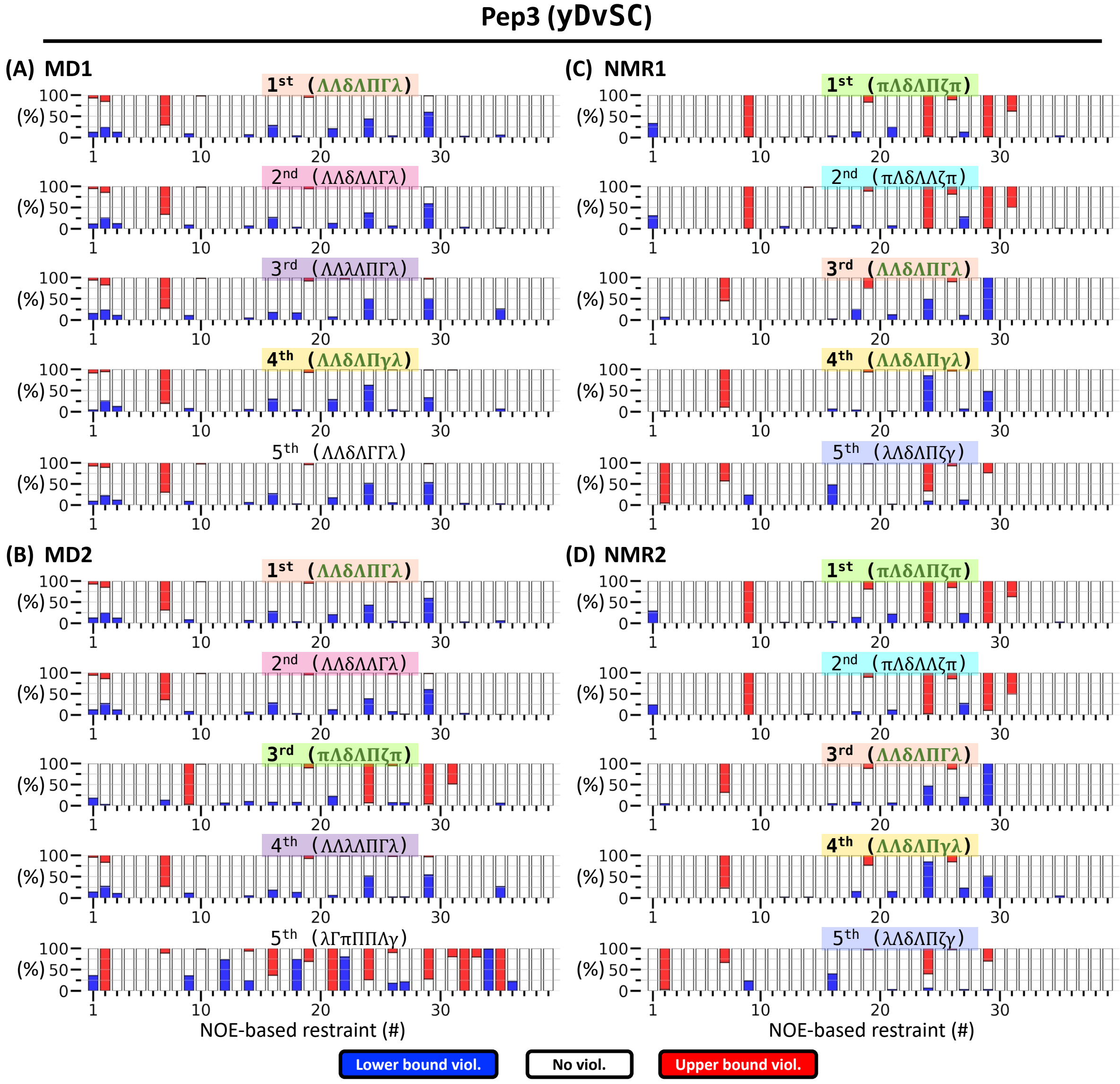

Figure S12. Violation analysis for the top five structures of peptide 3 (yDvSC). The percentages of frames with lower-bound and upper-bound violations are colored in blue and red, respectively. Panels (A)–(D) show the violation patterns from MD1, MD2, NMR1, and NMR2, respectively. The structural digit strings are colored as in Table 2. See Table S8 for the NOE-based distance restraints.

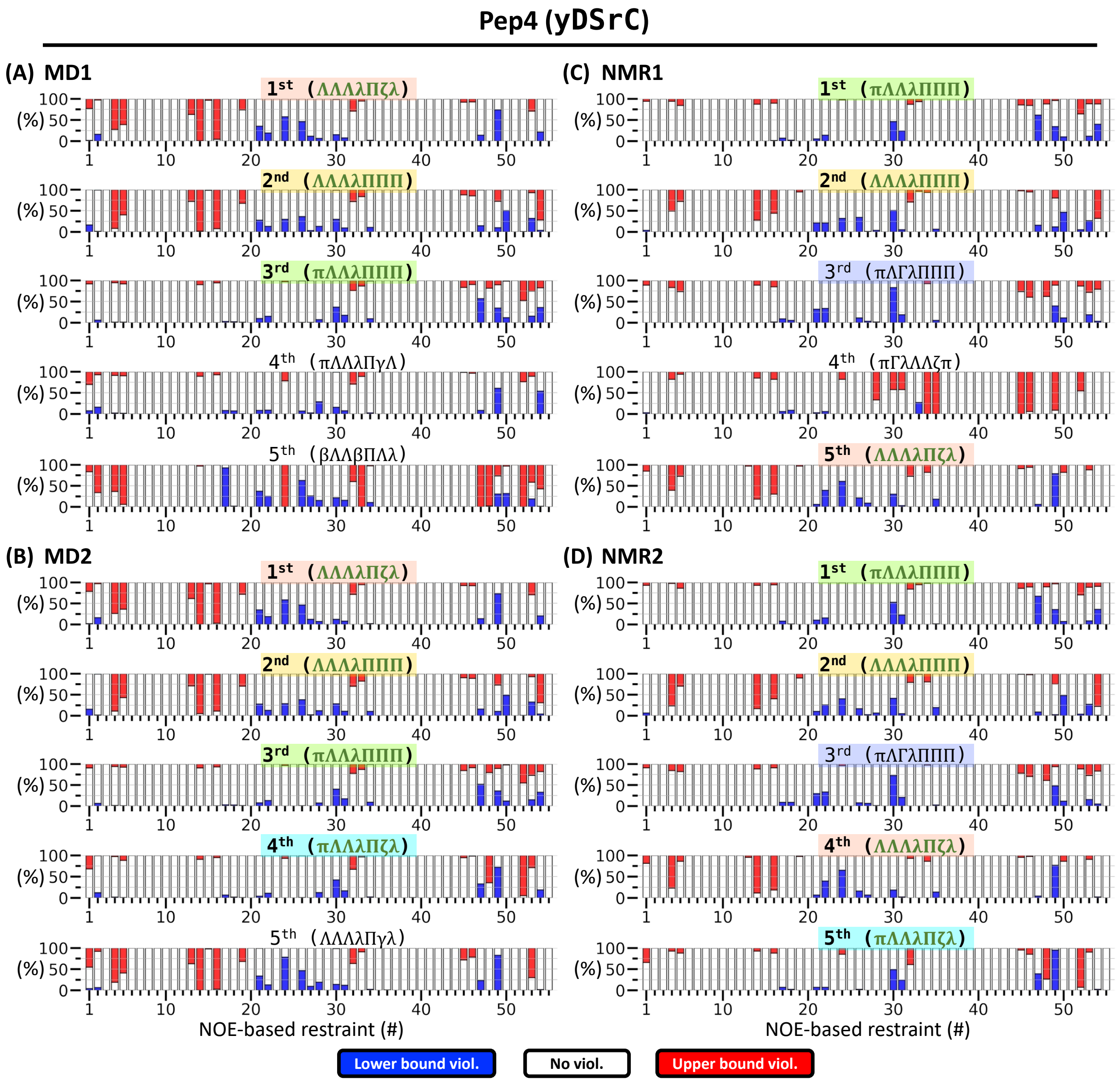

Figure S13. Violation analysis for the top five structures of peptide 4 (yDSrC). The percentages of frames with lower-bound and upper-bound violations are colored in blue and red, respectively. Panels (A)–(D) show the violation patterns from MD1, MD2, NMR1, and NMR2, respectively. The structural digit strings are colored as in Table 2. See Table S9 for the NOE-based distance restraints.

Table S1. A summary of the number of sequences converged within 50–100 ns, 100–200 ns, 200–300 ns, or 100–300 ns and the number of sequences that failed to converge for the training and test data sets, and the 31 additional sequences in **Table 1**.

	Converged					Not converged	Total
	50–100 ns	100–200 ns	200–300 ns	100–300 ns	Total		
Training set	177	158	44	17	396	20	416
Test set	28	18	4	0	50	3	53
31 additional in Table 1	20	7	3	0	30	1	31
Total	225	183	51	17	476	24	500

Table S2. Chemical shifts for peptide 1 (yVssC) at pH 4.91 (20 °C). Note: DTY is D-Tyr, DSE is D-Ser, and CYL is Cys with thioether bond.

Pep1 (yVssC)

Residue	NH	αH	βH	Other
DTY_1	8.38 (125.1)	4.67 (57.5)	2.90, 3.01 (39.7)	δ: 7.12 (133.2) ε: 6.85 (118.3)
VAL_2	8.30 (127.1)	3.74 (63.6)	1.87 (32.6)	*γ1: 0.81 (20.7) γ2: 0.73 (20.8)*
DSE_3	8.92 (122.0)	4.49 (58.7)	4.00, 3.89 (63.3)	
DSE_4	8.26 (116.7)	4.50 (59.0)	3.98, 3.98 (63.7)	
CYL_5	7.87 (121.1)	4.56 (54.8)	*β1: 3.11 β2: 2.67* (35.8)	*δ1: 3.46 δ2: 3.24* (37.15)
NH2_6	*H1: 7.48, H2: 7.19* (108.3)			

Stereospecific assignments are in italics. ^{13}C and ^{15}N assignments are in parentheses.

Table S3. Chemical shifts for peptide 2 (yavnC) at pH 4.77 (20 °C). Note: DTY is D-Tyr, DAL is D-Ala, DVA is D-Val, DSN is D-Asn, and CYL is Cys with thioether bond.

Pep2 (yavnC)

Residue	NH	αH	βH	Other
DTY_1	8.39 (126.8)	4.48 (59.5)	*β1: 3.10, β2: 2.99* (38.4)	δ: 7.16 (133.1) ε: 6.85 (118.2)
DAL_2	8.12 (122.5)	4.47 (51.0)	1.42 (20.2)	
DVA_3	8.29 (119.4)	3.90 (64.0)	2.08 (31.7)	*γ1:1.01 (20.5) γ2:0.93 (21.7)*
DSN_4	8.73 (119.5)	4.55 (53.9)	2.92, 2.99 (37.5)	*δ2: 6.89 δ1:7.63* (133.0)
CYL_5	8.15 (119.6)	4.35 (56.3)	3.04, 3.11 (34.0)	*δ1: 3.42 δ2:3.24* (38.03)
NH2_6	*H1: 7.52, H2: 7.34* (109.9)			

Stereospecific assignments are in italics. ^{13}C and ^{15}N assignments are in parentheses.

Table S4. Chemical shifts for peptide 3 (yDvSC) at pH 4.77 (20 °C). Note: DTY is D-Tyr, DVA is D-Val, and CYL is Cys with thioether bond.

Pep3 (yDvSC)

Residue	NH	αH	βH	Other
DTY_1	8.67 (123.6)	4.32 (59.6)	3.08, 3.08 (37.3)	δ: 7.14 (133.5) ε: 6.84 (118.1)
ASP_2	8.32 (124.1)	4.42 (53.5)	*β2: 2.83, β1: 2.41* (39.7)	
DVA_3	8.04 (114.6)	4.57 (60.7)	2.44 (31.8)	γ1: 0.90 (19.3) γ2: 0.89 (21.4)
SER_4	8.34 (118.8)	4.32 (59.9)	*β1: 3.91, β2: 3.98* (63.5)	
CYL_5	8.99 (122.8)	4.49 (57.9)	3.28, 2.73 (34.5)	*δ1: 3.60 δ2: 3.25* (36.3)
NH2_6	7.33, 7.31 (107.6)			

Stereospecific assignments are in italics. ^{13}C and ^{15}N assignments are in parentheses.

Table S5. Chemical shifts for peptide 4 (yDSrC) at pH 4.91 (20 °C). Note: DTY is D-Tyr, DAR is D-Arg, and CYL is Cys with thioether bond.

Pep4 (yDSrC)

Residue	NH	αH	βH	Other
DTY_1	8.64 (126.6)	4.44 (59.3)	2.97, 3.08 (38.0)	δ: 7.13 (133.5)<br>ε: 6.85 (118.4)
ASP_2	8.39 (125.1)	4.55 (53.9)	2.62, 2.40 (39.9)	
SER_3	8.16 (113.9)	4.52 (58.4)	4.01, 3.82 (63.9)	
DAR_4	8.10 (122.9)	4.34 (56.9)	1.91, 1.80 (30.6)	δ1: 3.21, δ2:3.21 (43.2)<br>ε:7.21 (81.9)<br>γ1:1.64 γ2:1.65 (27.2)
CYL_5	8.29 (118.9)	4.33 (56.3)	*β2: 2.96, β1: 3.18* (35.5)	*δ2: 3.42 δ1:3.20* (37.2)
NH2_6	*H1: 7.69, H2: 7.17* (108.6)			

Stereospecific assignments are in italics. ^{13}C and ^{15}N assignments are in parentheses.

Table S6. NOE-based distance restraints and violation statistics for peptide 1 (yVssC) from MD1, MD2, NMR1, and NMR2. Note that DTY is D-Tyr, DSE is D-Ser, and CYL is Cys with thioether bond. Res 1, a 1, res 2, and a 2 are residue and atom information of each proton pair. 'lb v' and 'up v' are lower bound and upper bound violations, respectively. The percentages of frames for lower-bound and upper-bound violations in each simulation are also included.

Pep1 (yVssC)														
	res 1	a 1	res 2	a 2	lb (Å)	up (Å)	MD1		MD2		NMR1		NMR2	
							lb v (%)	ub v (%)	lb v (%)	ub v (%)	lb v (%)	ub v (%)	lb v (%)	ub v (%)
1	DTY_1	H	CYL_5	HD2	2.914	4.372	23.22	0.00	17.10	0.00	37.00	0.00	38.93	0.00
2	DTY_1	H	CYL_5	HD1	2.358	3.538	24.88	10.12	19.25	7.40	31.25	20.87	27.91	19.92
3	DTY_1	HD#	CYL_5	HD1	3.951	7.927	0.00	0.00	0.00	0.00	0.00	0.00	0.00	0.00
4	DTY_1	H	DTY_1	HA	1.991	2.987	0.01	17.53	0.00	16.36	0.00	5.24	0.00	5.56
5	DTY_1	H	DTY_1	HB2	2.781	5.171	26.37	0.00	19.67	0.00	39.21	0.00	39.84	0.00
6	DTY_1	HB2	DTY_1	HD#	2.342	5.512	0.00	0.00	0.00	0.00	0.00	0.00	0.00	0.00
7	DTY_1	HB1	DTY_1	HD#	2.269	5.403	0.00	0.00	0.00	0.00	0.00	0.00	0.00	0.00
8	DTY_1	H	DTY_1	HB1	2.625	4.937	23.18	0.00	16.14	0.00	39.52	0.00	39.13	0.00
9	DTY_1	HD#	DTY_1	HE#	1.762	4.644	0.00	0.00	0.00	0.00	0.00	0.00	0.00	0.00
10	DTY_1	HB1	DTY_1	HE#	3.494	6.240	0.00	0.00	0.00	0.00	0.00	0.00	0.00	0.00
11	DTY_1	HB2	DTY_1	HE#	3.531	6.297	0.00	0.00	0.00	0.00	0.00	0.00	0.00	0.00
12	DTY_1	HB1	VAL_2	H	2.602	7.070	5.22	0.00	3.68	0.00	10.18	0.00	9.40	0.00
13	DTY_1	HB2	VAL_2	H	2.724	7.128	3.68	0.00	2.44	0.00	4.33	0.00	4.85	0.00
14	DTY_1	HA	DTY_1	HD#	2.488	4.732	0.32	1.57	0.28	1.76	0.00	0.30	0.00	0.10
15	DTY_1	HA	DTY_1	HE#	3.821	6.731	0.00	0.00	0.00	0.00	0.00	0.00	0.00	0.00
16	DTY_1	H	DTY_1	HD#	3.138	6.706	0.00	0.00	0.00	0.00	0.00	0.00	0.00	0.00
17	DTY_1	HA	VAL_2	H	2.006	3.010	5.11	2.35	4.67	1.23	0.00	6.05	0.00	4.25
18	DTY_1	HD#	VAL_2	HG2#	3.142	7.712	0.00	0.90	0.00	1.20	0.00	2.02	0.00	1.92
19	DTY_1	HD#	VAL_2	HG1#	3.699	8.549	0.00	3.93	0.00	3.78	0.00	0.00	0.00	0.10
20	DTY_1	HD#	VAL_2	HA	3.148	6.722	0.25	4.95	0.34	3.78	1.41	3.43	1.01	3.44
21	DTY_1	HD#	VAL_2	H	3.103	6.655	0.00	0.00	0.00	0.00	0.00	0.00	0.00	0.00
22	DTY_1	H	CYL_5	HB2	2.935	5.403	5.79	5.26	4.33	3.62	10.48	3.83	8.70	3.03
23	DTY_1	H	CYL_5	HB1	3.654	6.480	10.31	0.01	6.35	0.00	12.70	0.00	11.43	0.00
24	VAL_2	HG1#	DTY_1	HE#	3.066	6.598	0.00	34.92	0.00	30.93	0.00	45.77	0.00	43.48
25	VAL_2	HG2#	DTY_1	HE#	4.142	8.214	0.00	5.30	0.01	5.00	0.00	7.66	0.00	6.67
26	VAL_2	HA	DTY_1	HE#	3.880	7.820	0.00	19.48	0.00	16.04	0.00	17.94	0.00	17.80
27	VAL_2	H	VAL_2	HB	2.341	3.511	12.96	18.30	14.13	10.68	1.11	36.39	1.31	33.97
28	VAL_2	H	VAL_2	HG2#	2.270	4.406	0.00	2.90	0.00	2.44	0.00	9.07	0.00	7.68
29	DSE_3	H	VAL_2	HG1#	2.844	5.266	0.00	0.08	0.00	0.07	0.00	0.00	0.00	0.00
30	VAL_2	H	VAL_2	HA	2.459	3.689	1.15	0.00	0.11	0.00	1.81	0.00	0.91	0.00
31	DSE_3	H	VAL_2	HB	3.286	4.928	12.27	0.00	11.76	0.00	18.75	0.00	17.90	0.00
32	DSE_3	H	VAL_2	HG2#	3.638	6.456	0.06	0.00	0.03	0.00	0.00	0.00	0.00	0.00
33	DSE_3	H	VAL_2	HG1#	2.914	5.372	0.00	0.01	0.00	0.00	0.00	0.00	0.00	0.00
34	DSE_3	H	VAL_2	HA	2.085	3.127	22.14	7.72	23.24	8.41	2.22	11.79	2.12	12.34
35	DSE_3	H	DSE_3	HA	2.432	3.648	1.69	0.00	2.17	0.00	10.89	0.00	9.91	0.00
36	DSE_3	H	DSE_3	HB#	2.786	5.178	2.94	0.00	1.73	0.00	2.42	0.00	1.82	0.00
37	DSE_3	H	DSE_3	HB#	2.789	5.183	2.97	0.00	1.75	0.00	2.42	0.00	1.82	0.00
38	DSE_3	H	DSE_4	H	2.470	3.706	30.08	16.37	33.50	10.12	28.43	8.37	28.51	8.19
39	DSE_4	HB#	DTY_1	HD#	3.799	9.699	0.00	17.75	0.00	12.71	0.00	11.69	0.00	13.04
40	DSE_4	HB#	DTY_1	HE#	4.051	10.077	0.04	24.80	0.02	18.45	0.00	22.18	0.00	22.65
41	DSE_4	H	VAL_2	HA	3.088	4.632	2.18	17.72	2.39	10.50	0.30	18.65	0.61	19.21
42	DSE_4	H	DSE_4	HA	2.077	3.115	0.38	0.08	0.07	0.08	0.00	0.00	0.00	0.00
43	DSE_4	H	DSE_4	HB#	2.456	4.684	0.20	0.00	0.18	0.00	0.00	0.00	0.00	0.00
44	CYL_5	HB2	NH2_6	H1	3.506	5.258	7.90	0.01	7.48	0.03	3.12	0.71	1.42	0.81
45	CYL_5	HB1	NH2_6	H1	3.254	4.882	38.66	0.76	41.74	0.36	27.32	1.81	26.49	3.24
46	CYL_5	H	CYL_5	HD1	3.890	5.836	1.54	2.72	1.31	2.12	4.13	0.71	3.54	1.21
47	CYL_5	HA	NH2_6	H1	2.606	3.908	51.12	11.89	53.12	11.52	34.48	26.01	32.25	22.75
48	CYL_5	HA	NH2_6	H2	3.304	4.956	16.35	0.00	16.92	0.00	24.40	0.00	26.29	0.00
49	CYL_5	H	NH2_6	H1	3.038	4.558	13.85	24.37	12.58	22.71	19.56	19.96	20.63	19.51
50	CYL_5	H	DSE_4	HB1	3.128	5.692	17.44	0.00	18.15	0.00	10.79	0.00	11.93	0.00
51	CYL_5	H	DSE_4	H	2.578	3.866	9.82	66.19	7.67	79.80	6.75	38.51	6.77	38.32
52	CYL_5	H	CYL_5	HA	2.806	4.210	14.84	0.00	7.30	0.00	17.64	0.00	20.93	0.00
53	CYL_5	H	CYL_5	HB2	2.506	4.758	26.88	0.00	23.46	0.00	39.72	0.00	38.62	0.00
54	CYL_5	H	CYL_5	HB1	3.144	5.716	5.86	0.00	3.55	0.00	5.24	0.00	4.45	0.00

Table S7. NOE-based distance restraints and violation statistics for peptide 2 (yavnC) from MD1, MD2, NMR1, and NMR2. Note that DTY is D-Tyr, DAL is D-Ala, DVA is D-Val, DSN is D-Asn, and CYL is Cys with thioether bond. Res 1, a 1, res 2, and a 2 are residue and atom information of each proton pair. 'lb v' and 'up v' are lower bound and upper bound violations, respectively. The percentages of frames for lower-bound and upper-bound violations in each simulation are also included.

Pep2 (yavnC)														
	res 1	a 1	res 2	a 2	lb (Å)	up (Å)	MD1		MD2		NMR1		NMR2	
							lb v (%)	ub v (%)	lb v (%)	ub v (%)	lb v (%)	ub v (%)	lb v (%)	ub v (%)
1	DTY_1	H	CYL_5	HD1	2.446	3.670	6.59	0.83	3.93	1.05	1.62	0.00	0.62	0.00
2	DTY_1	H	CYL_5	HD2	1.840	2.760	0.21	7.90	0.17	6.20	0.00	8.39	0.00	7.17
3	DTY_1	H	DTY_1	HA	2.206	3.308	3.08	0.00	2.03	0.00	0.61	0.00	0.42	0.00
4	DTY_1	H	DTY_1	HB2	2.050	3.074	0.76	26.20	0.78	28.87	0.00	16.18	0.00	20.69
5	DTY_1	H	DTY_1	HB1	2.232	3.348	3.88	32.46	4.51	34.66	0.40	41.35	0.73	36.38
6	DTY_1	HB2	DTY_1	HD#	1.950	4.926	0.00	0.00	0.00	0.00	0.00	0.00	0.00	0.00
7	DTY_1	HB1	DTY_1	HE#	2.987	6.481	0.00	0.00	0.00	0.00	0.00	0.00	0.00	0.00
8	DTY_1	HA	DTY_1	HD#	2.022	5.032	0.00	0.00	0.00	0.00	0.00	0.00	0.00	0.00
9	DTY_1	H	DTY_1	HD#	2.366	5.548	0.01	0.00	0.00	0.00	0.00	0.00	0.00	0.00
10	DTY_1	HB2	DTY_1	HE#	2.874	6.312	0.00	0.00	0.00	0.00	0.00	0.00	0.00	0.00
11	DTY_1	HB1	DTY_1	HD#	1.960	4.940	0.00	0.00	0.00	0.00	0.00	0.00	0.00	0.00
12	DTY_1	HA	DTY_1	HE#	3.100	6.650	0.00	0.00	0.00	0.00	0.00	0.00	0.00	0.00
13	DTY_1	H	DAL_2	H	2.010	3.014	3.01	16.43	3.28	13.27	1.72	9.20	1.04	21.00
14	DTY_1	HB1	DAL_2	H	2.177	4.265	0.75	7.62	0.77	7.56	0.00	4.75	0.00	11.02
15	DTY_1	HB2	DAL_2	H	2.395	3.593	8.23	28.11	7.23	29.10	1.11	26.59	2.08	34.82
16	DAL_2	HB#	NH2_6	H1	2.998	5.496	0.00	23.35	0.00	25.17	0.00	14.96	0.00	26.61
17	DAL_2	HB#	NH2_6	H2	2.601	4.901	0.00	25.66	0.00	27.92	0.00	12.94	0.00	27.75
18	DAL_2	H	CYL_5	HD2	3.215	4.823	0.82	6.28	0.79	7.14	0.10	18.10	0.10	25.05
19	DAL_2	H	NH2_6	H1	3.282	4.922	0.76	67.92	0.49	72.82	1.11	62.39	2.81	59.04
20	DAL_2	HB#	DTY_1	HD#	2.782	8.174	0.00	0.00	0.00	0.00	0.00	0.00	0.00	0.00
21	DAL_2	H	DTY_1	HD#	3.034	5.550	0.68	1.48	0.59	1.69	0.20	0.30	0.10	0.10
22	DAL_2	HB#	DTY_1	HE#	2.956	8.434	0.00	28.29	0.00	38.16	0.00	27.70	0.00	26.20
23	DAL_2	HA	DAL_2	H	2.212	3.318	1.04	0.00	2.13	0.00	0.20	0.00	0.73	0.00
24	DAL_2	HB#	DAL_2	H	1.973	3.959	0.00	0.00	0.00	0.00	0.00	0.00	0.00	0.00
25	DVA_3	H	DAL_2	HA	1.998	2.998	0.33	6.05	0.24	7.30	0.00	2.43	0.00	16.11
26	DVA_3	H	DAL_2	HB#	2.064	4.096	0.00	1.45	0.00	2.58	0.00	21.64	0.00	17.88
27	DVA_3	HA	DVA_3	H	2.259	3.389	3.44	0.00	1.90	0.00	25.38	0.00	23.08	0.00
28	DVA_3	H	DVA_3	HB	2.712	4.068	67.01	0.28	63.62	0.12	46.41	0.00	46.05	0.00
29	DVA_3	H	DVA_3	HG1#	2.203	4.305	0.00	8.32	0.00	12.61	0.00	17.69	0.00	17.05
30	DVA_3	H	DVA_3	HG2#	2.004	4.006	0.00	73.25	0.00	61.88	0.00	56.52	0.00	56.34
31	DVA_3	HG1#	DSN_4	HD21	2.899	6.349	0.00	62.64	0.00	56.43	0.00	49.34	0.00	50.83
32	DVA_3	HG2#	DSN_4	HD21	3.410	7.114	0.42	10.99	0.37	14.09	0.00	14.16	0.00	14.03
33	DVA_3	HG1#	DSN_4	HD22	3.031	6.547	0.00	55.77	0.00	51.31	0.00	46.61	0.00	48.13
34	DVA_3	HG2#	DSN_4	HD22	3.279	6.919	0.08	14.68	0.06	17.85	0.00	21.33	0.00	21.21
35	DVA_3	HA	CYL_5	H	3.358	5.038	4.60	1.96	3.81	2.52	0.00	0.71	0.52	2.60
36	DSN_4	H	DVA_3	HA	2.469	3.703	85.03	0.14	69.61	0.62	15.67	0.00	13.93	0.00
37	DSN_4	H	DVA_3	HG1#	3.238	5.856	0.00	0.00	0.02	0.00	0.00	0.00	0.00	0.00
38	DSN_4	H	DVA_3	HG2#	3.442	6.164	0.01	0.00	0.04	0.00	0.00	0.00	0.00	0.00
39	DSN_4	H	DSN_4	HA	2.630	3.944	92.60	0.00	74.12	0.00	16.89	0.00	15.07	0.00
40	DSN_4	H	DSN_4	HB2	2.510	6.264	1.48	0.00	4.89	0.00	7.99	0.00	7.59	0.00
41	DSN_4	H	DSN_4	HB1	2.890	6.836	7.67	0.00	12.25	0.00	13.95	0.00	20.58	0.00
42	DSN_4	HB2	DSN_4	HD21	2.115	4.173	2.56	0.17	2.71	0.18	0.10	0.10	0.00	0.00
43	DSN_4	HB2	DSN_4	HD22	2.672	5.008	4.65	0.00	4.51	0.00	8.49	0.00	8.52	0.00
44	DSN_4	HB1	DSN_4	HD22	2.849	5.273	0.66	0.00	1.07	0.00	7.38	0.00	8.21	0.00
45	DSN_4	HA	DSN_4	HD21	3.007	4.511	5.51	19.84	5.13	21.04	1.92	45.40	1.77	44.91
46	DSN_4	HA	CYL_5	H	2.898	4.348	29.63	0.00	27.47	0.00	14.76	0.00	25.26	0.00
47	CYL_5	HA	NH2_6	H1	2.296	3.444	15.54	38.29	19.85	32.37	14.46	35.89	17.46	31.39
48	CYL_5	H	NH2_6	H1	3.147	4.721	51.91	10.26	43.18	13.00	30.54	6.37	25.05	4.47
49	CYL_5	HA	NH2_6	H2	3.022	4.534	0.02	0.00	0.02	0.00	8.09	0.00	10.91	0.00
50	CYL_5	H	DAL_2	HB#	3.276	5.914	0.00	7.12	0.00	8.42	0.00	0.81	0.00	11.75
51	CYL_5	H	CYL_5	HA	2.584	3.876	1.48	0.00	1.66	0.00	0.20	0.00	6.13	0.00
52	CYL_5	H	CYL_5	HB2	2.552	3.828	76.75	0.04	78.35	0.07	73.41	0.91	62.06	0.62
53	CYL_5	H	CYL_5	HB1	2.553	3.829	5.08	2.34	4.69	2.64	16.89	8.19	11.12	13.41

Table S8. NOE-based distance restraints and violation statistics for peptide 3 (yDvSC) from MD1, MD2, NMR1, and NMR2. Note that DTY is D-Tyr, DVA is D-Val, and CYL is Cys with thioether bond. Res 1, a 1, res 2, and a 2 are residue and atom information of each proton pair. 'lb v' and 'up v' are lower bound and upper bound violations, respectively. The percentages of frames for lower-bound and upper-bound violations in each simulation are also included.

Pep3 (yDvSC)														
	res 1	a 1	res 2	a 2	lb (Å)	up (Å)	MD1		MD2		NMR1		NMR2	
							lb v (%)	ub v (%)	lb v (%)	ub v (%)	lb v (%)	ub v (%)	lb v (%)	ub v (%)
1	DTY_1	H	CYL_5	HD1	2.377	3.565	13.40	8.29	15.84	7.92	17.72	5.40	16.49	7.17
2	DTY_1	H	CYL_5	HD2	2.118	3.178	18.60	18.62	16.02	20.18	2.04	13.34	1.18	13.38
3	DTY_1	H	DTY_1	HA	2.135	3.203	8.14	0.00	5.75	0.00	0.00	0.00	0.00	0.00
4	DTY_1	HD#	DTY_1	HB#	0.943	4.915	0.00	0.00	0.00	0.00	0.00	0.00	0.00	0.00
5	DTY_1	HB#	DTY_1	HE#	1.919	6.379	0.00	0.00	0.00	0.00	0.00	0.00	0.00	0.00
6	DTY_1	H	DTY_1	HD#	1.895	5.343	0.00	0.00	0.00	0.00	0.00	0.00	0.00	0.00
7	DTY_1	HA	ASP_2	H	1.997	2.995	0.86	54.59	1.76	44.17	0.00	19.45	0.11	18.74
8	DTY_1	H	DVA_3	HG#	2.749	7.623	0.00	7.44	0.00	9.97	0.00	0.71	0.00	2.25
9	ASP_2	H	CYL_5	HD1	3.491	5.237	7.76	16.07	6.86	26.91	3.67	43.58	3.85	47.43
10	ASP_2	H	DTY_1	HD#	3.150	5.726	0.10	1.53	0.20	1.39	0.00	0.00	0.00	0.21
11	ASP_2	H	ASP_2	HA	1.714	4.072	0.00	0.00	0.00	0.00	0.00	0.00	0.00	0.00
12	ASP_2	H	ASP_2	HB2	2.878	4.316	16.55	0.00	20.85	0.00	3.46	0.00	3.10	0.00
13	ASP_2	H	DTY_1	HB#	3.169	5.753	0.08	0.00	0.56	0.00	0.00	0.00	0.00	0.00
14	ASP_2	H	ASP_2	HB1	2.447	3.671	10.88	4.42	12.81	7.18	0.81	0.71	1.82	1.07
15	DVA_3	H	DTY_1	HB#	2.490	6.234	0.00	7.98	0.00	9.00	0.00	0.92	0.00	3.10
16	DVA_3	H	ASP_2	H	2.400	3.600	19.62	19.82	15.45	22.86	15.27	1.32	14.13	3.75
17	DVA_3	H	DVA_3	HA	2.690	4.034	2.09	0.00	3.40	0.00	1.63	0.00	4.18	0.00
18	DVA_3	H	DVA_3	HB	3.118	4.676	17.58	0.00	23.33	0.00	12.42	0.00	11.13	0.00
19	DVA_3	H	DVA_3	HG1#	1.274	4.410	0.00	12.08	0.00	15.22	0.00	12.93	0.00	13.70
20	DVA_3	H	DVA_3	HG2#	1.474	4.712	0.00	0.25	0.00	0.53	0.00	0.10	0.00	0.21
21	DVA_3	H	SER_4	H	2.134	3.200	13.75	22.16	12.28	28.46	8.04	1.32	10.17	2.25
22	DVA_3	HA	SER_4	H	2.363	3.545	17.17	1.01	22.33	1.52	1.22	0.10	2.36	0.43
23	DVA_3	HG#	SER_4	H	2.058	6.588	0.00	0.00	0.00	0.00	0.00	0.00	0.00	0.00
24	SER_4	H	CYL_5	HD1	3.521	5.281	28.78	28.91	21.60	41.12	11.00	55.30	10.81	57.60
25	SER_4	H	SER_4	HA	1.841	4.261	0.00	0.00	0.00	0.00	0.00	0.00	0.00	0.00
26	SER_4	H	SER_4	HB1	2.405	3.607	8.74	5.60	10.02	6.81	1.63	11.91	0.86	13.49
27	SER_4	H	SER_4	HB2	2.712	4.068	7.60	0.56	10.07	0.51	14.56	0.00	18.20	0.00
28	CYL_5	H	NH2_6	H#	1.660	4.990	0.00	0.03	0.00	0.05	0.00	0.00	0.00	0.00
29	CYL_5	H	CYL_5	HD1	2.540	3.810	38.11	31.09	28.54	44.72	17.72	57.84	15.31	60.06
30	CYL_5	HB2	DTY_1	H	2.463	6.195	1.20	0.13	0.95	0.33	0.10	0.92	0.32	1.71
31	CYL_5	HB1	DTY_1	HD#	0.859	6.289	0.00	19.01	0.00	28.23	0.00	30.35	0.00	29.87
32	CYL_5	H	DVA_3	H	2.941	4.411	2.26	25.25	1.63	31.39	0.71	7.03	0.64	9.21
33	CYL_5	H	DVA_3	HG#	1.822	6.232	0.00	5.26	0.00	5.90	0.00	0.00	0.00	0.21
34	CYL_5	H	SER_4	HA	2.651	3.977	19.64	0.00	25.19	0.00	4.68	0.00	5.57	0.00
35	CYL_5	H	SER_4	H	2.354	3.532	7.25	19.58	7.60	25.14	13.14	4.38	9.96	4.60
36	CYL_5	H	SER_4	HB2	2.490	5.234	2.15	0.00	2.71	0.00	0.41	0.00	0.43	0.00
37	CYL_5	H	CYL_5	HA	1.349	3.523	0.00	0.00	0.00	0.00	0.00	0.00	0.00	0.00
38	CYL_5	H	CYL_5	HB2	1.549	4.823	0.00	0.00	0.00	0.00	0.00	0.00	0.00	0.00
39	CYL_5	H	CYL_5	HB1	1.439	4.659	0.00	0.00	0.00	0.00	0.00	0.00	0.00	0.00

Table S9. NOE-based distance restraints and violation statistics for peptide 4 (yDSrC) from MD1, MD2, NMR1, and NMR2. Note that DTY is D-Tyr, DAR is D-Arg, and CYL is Cys with thioether bond. Res 1, a 1, res 2, and a 2 are residue and atom information of each proton pair. 'lb v' and 'up v' are lower bound and upper bound violations, respectively. The percentages of frames for lower-bound and upper-bound violations in each simulation are also included.

Pep4 (yDSrC)														
	res 1	a 1	res 2	a 2	lb (Å)	up (Å)	MD1		MD2		NMR1		NMR2	
							lb v (%)	ub v (%)	lb v (%)	ub v (%)	lb v (%)	ub v (%)	lb v (%)	ub v (%)
1	DTY_1	H	CYL_5	HD1	2.310	3.466	6.73	20.60	5.99	20.19	1.73	21.17	1.47	15.81
2	DTY_1	H	CYL_5	HD2	2.042	3.064	7.87	10.41	8.16	10.23	0.00	3.68	0.12	1.47
3	DTY_1	H	DTY_1	HA	1.274	3.412	0.00	0.00	0.00	0.00	0.00	0.00	0.00	0.00
4	DTY_1	H	DTY_1	HB2	2.117	3.425	0.64	55.99	0.70	50.72	0.00	18.99	0.00	19.24
5	DTY_1	H	DTY_1	HB1	2.162	3.492	0.46	49.87	0.57	46.60	0.00	24.17	0.00	18.63
6	DTY_1	HB2	DTY_1	HD#	2.138	5.208	0.00	0.00	0.00	0.00	0.00	0.00	0.00	0.00
7	DTY_1	HB1	DTY_1	HD#	2.097	5.145	0.00	0.00	0.00	0.00	0.00	0.00	0.00	0.00
8	DTY_1	HB1	DTY_1	HE#	3.131	6.697	0.00	0.00	0.00	0.00	0.00	0.00	0.00	0.00
9	DTY_1	HB2	DTY_1	HE#	3.171	6.757	0.00	0.00	0.00	0.00	0.00	0.00	0.00	0.00
10	DTY_1	HA	DTY_1	HD#	2.234	5.352	0.00	0.00	0.00	0.00	0.00	0.00	0.00	0.00
11	DTY_1	HA	DTY_1	HE#	3.273	6.909	0.00	0.00	0.00	0.00	0.00	0.00	0.00	0.00
12	DTY_1	H	DTY_1	HD#	2.698	6.048	0.00	0.00	0.00	0.00	0.00	0.00	0.00	0.00
13	ASP_2	HB1	DTY_1	HD#	3.125	8.287	0.00	16.75	0.00	15.03	0.00	0.12	0.00	0.25
14	ASP_2	HB1	DTY_1	HE#	2.836	7.854	0.00	65.50	0.00	60.07	0.00	25.66	0.00	22.30
15	ASP_2	HB2	DTY_1	HD#	3.866	8.798	0.01	1.36	0.00	1.14	0.00	0.00	0.00	0.00
16	ASP_2	HB2	DTY_1	HE#	3.929	8.893	0.18	55.86	0.20	50.55	0.69	20.14	0.49	18.63
17	ASP_2	H	DTY_1	HB1	3.054	5.582	13.22	0.00	12.22	0.00	7.94	0.00	9.56	0.00
18	ASP_2	H	DTY_1	HB2	3.105	5.657	5.36	0.00	5.40	0.00	5.06	0.00	4.29	0.00
19	ASP_2	H	DTY_1	HA	1.888	2.832	0.27	16.99	0.39	16.47	0.00	7.02	0.00	2.21
20	ASP_2	H	DTY_1	HD#	2.930	6.394	0.00	0.00	0.00	0.00	0.00	0.00	0.00	0.00
21	ASP_2	H	ASP_2	HB2	2.461	3.991	26.89	0.40	24.48	0.91	7.83	0.00	12.87	0.00
22	ASP_2	H	ASP_2	HB1	2.649	4.273	18.54	0.00	17.32	0.01	18.87	0.00	22.43	0.00
23	ASP_2	H	ASP_2	HA	2.462	3.692	1.03	0.00	2.17	0.00	0.35	0.00	0.86	0.00
24	SER_3	H	CYL_5	HD1	3.762	5.644	24.00	25.53	20.40	27.61	5.75	12.08	7.72	9.07
25	ASP_2	HA	DTY_1	HD#	2.976	7.464	0.00	0.00	0.00	0.00	0.00	0.00	0.00	0.00
26	SER_3	H	ASP_2	HB2	3.112	4.768	33.58	0.18	32.86	0.23	6.90	0.00	5.64	0.00
27	SER_3	H	ASP_2	HB1	3.190	4.886	9.74	0.04	9.05	0.05	1.84	0.00	2.70	0.00
28	SER_3	H	ASP_2	H	2.258	3.388	10.99	5.80	10.29	8.27	2.76	9.90	3.06	1.84
29	SER_3	H	SER_3	HA	2.009	4.013	0.01	0.00	0.02	0.00	0.00	0.00	0.00	0.00
30	SER_3	H	SER_3	HB2	2.574	3.962	23.05	2.38	23.73	2.93	29.57	10.01	44.24	0.74
31	SER_3	H	SER_3	HB1	2.644	4.066	11.29	1.51	13.30	1.77	13.35	9.90	20.22	0.49
32	SER_3	HA	DAR_4	H	2.298	3.446	5.19	25.98	7.39	25.01	5.18	18.18	5.64	16.18
33	SER_3	HA	CYL_5	H	3.602	5.404	2.46	19.71	3.63	19.04	9.21	2.65	0.61	4.29
34	CYL_5	HA	NH2_6	H1	2.151	3.227	5.88	8.68	5.75	11.26	0.00	18.99	0.12	12.38
35	CYL_5	HA	NH2_6	H2	2.674	4.010	0.01	4.14	0.03	5.76	2.88	11.97	4.04	4.29
36	DAR_4	H	DAR_4	HG#	2.557	4.835	0.21	0.06	0.14	0.11	0.00	0.35	0.00	0.00
37	DAR_4	H	DAR_4	HB2	1.323	4.485	0.00	0.00	0.00	0.00	0.00	0.00	0.00	0.00
38	DAR_4	H	DAR_4	HB1	1.641	4.961	0.00	0.00	0.00	0.00	0.00	0.00	0.00	0.00
39	DAR_4	HB1	DAR_4	HE	1.955	5.433	0.45	0.02	0.44	0.02	0.00	0.00	0.12	0.00
40	DAR_4	H	DAR_4	HA	2.146	3.22	0.44	0.00	0.58	0.00	0.00	0.00	0.00	0.00
41	DAR_4	HE	DAR_4	HD#	1.232	4.348	0.00	0.00	0.00	0.00	0.00	0.00	0.00	0.00
42	DAR_4	HG#	DAR_4	HE	1.650	4.974	0.00	0.00	0.00	0.00	0.00	0.00	0.00	0.00
43	DAR_4	HB2	DAR_4	HE	2.022	5.532	0.39	0.01	0.34	0.00	0.12	0.00	0.25	0.00
44	DAR_4	HG#	CYL_5	H	2.301	5.951	0.00	0.00	0.00	0.00	0.00	0.00	0.00	0.00
45	DAR_4	HB2	CYL_5	H	1.834	4.252	0.08	15.66	0.10	17.71	0.00	41.66	0.00	19.24
46	DAR_4	HB1	CYL_5	H	1.746	4.120	0.02	12.63	0.05	13.50	0.00	34.52	0.00	14.58
47	DAR_4	H	CYL_5	H	2.127	3.191	15.15	16.28	16.99	15.72	21.86	6.56	35.91	2.33
48	CYL_5	H	CYL_5	HD2	1.641	4.961	0.00	22.36	0.00	26.31	0.00	16.80	0.00	20.34
49	CYL_5	HB2	NH2_6	H1	2.738	4.106	38.81	17.20	39.23	17.34	37.17	18.64	39.83	10.78
50	CYL_5	HB1	NH2_6	H1	3.018	4.528	18.05	4.13	16.50	4.36	15.77	4.95	11.64	3.92
51	CYL_5	H	NH2_6	H1	1.978	5.466	0.20	0.00	0.19	0.01	0.00	0.00	0.00	0.00
52	CYL_5	H	DTY_1	HB2	2.723	6.585	0.21	33.47	0.12	41.07	0.46	36.82	0.37	28.92
53	CYL_5	H	CYL_5	HB2	2.410	3.614	14.03	24.00	14.37	24.95	17.15	14.84	12.99	14.71
54	CYL_5	H	CYL_5	HB1	2.290	3.434	15.01	36.40	14.50	34.95	13.12	31.19	19.49	27.08
55	CYL_5	H	CYL_5	HA	2.156	3.234	0.87	0.00	0.86	0.00	0.00	0.00	0.12	0.00

Table S10. Comparison of amino acid chirality pattern versus structural digit chirality pattern for predicted top structures of the entire sequence space (3,375 sequences) at position R2.

Position R2

Amino acids \ Structural digits	Β	Π	Γ	Λ	β	π	γ	λ	δ
A	0.02	0.36	0.17	0.45	0.00	0.00	0.00	0.00	0.00
V	0.00	0.50	0.00	0.50	0.00	0.00	0.00	0.00	0.00
F	0.33	0.48	0.01	0.18	0.00	0.00	0.00	0.00	0.00
N	0.20	0.17	0.20	0.43	0.00	0.00	0.00	0.00	0.00
S	0.20	0.40	0.00	0.40	0.00	0.00	0.00	0.00	0.00
D	0.06	0.16	0.16	0.59	0.00	0.00	0.02	0.01	0.00
R	0.00	0.29	0.12	0.59	0.00	0.00	0.00	0.00	0.00
G	0.00	0.05	0.00	0.41	0.16	0.34	0.00	0.04	0.00
a	0.00	0.00	0.00	0.00	0.59	0.37	0.01	0.02	0.01
v	0.00	0.00	0.04	0.00	0.59	0.27	0.00	0.04	0.06
f	0.00	0.00	0.00	0.01	0.57	0.36	0.00	0.04	0.02
n	0.00	0.00	0.02	0.16	0.25	0.25	0.00	0.03	0.29
s	0.00	0.00	0.01	0.00	0.53	0.41	0.00	0.04	0.01
d	0.00	0.00	0.00	0.00	0.26	0.34	0.06	0.10	0.24
r	0.00	0.00	0.09	0.22	0.50	0.17	0.00	0.01	0.01

Table S11. Comparison of amino acid chirality pattern versus structural digit chirality pattern for predicted top structures of the entire sequence space (3,375 sequences) at position R3.

Position R3

Structural digits (columns) vs. Amino acids (rows); Fraction (0.0–1.0)

Amino acids	Β	Π	Γ	Λ	β	π	γ	λ	δ
A	0.09	0.41	0.12	0.38	0.00	0.00	0.00	0.00	0.00
V	0.36	0.39	0.00	0.25	0.00	0.00	0.00	0.00	0.00
F	0.40	0.34	0.04	0.22	0.00	0.00	0.00	0.00	0.00
N	0.04	0.39	0.25	0.30	0.00	0.00	0.00	0.02	0.00
S	0.05	0.42	0.02	0.51	0.00	0.00	0.00	0.00	0.00
D	0.00	0.24	0.22	0.54	0.00	0.00	0.00	0.00	0.00
R	0.39	0.34	0.04	0.23	0.00	0.00	0.00	0.00	0.00
G	0.07	0.43	0.01	0.19	0.07	0.06	0.00	0.17	0.00
a	0.00	0.00	0.01	0.00	0.18	0.54	0.00	0.25	0.02
v	0.00	0.00	0.08	0.00	0.08	0.53	0.00	0.15	0.16
f	0.00	0.00	0.00	0.00	0.16	0.64	0.00	0.17	0.03
n	0.00	0.00	0.01	0.48	0.11	0.21	0.01	0.16	0.02
s	0.00	0.00	0.00	0.00	0.31	0.36	0.00	0.33	0.00
d	0.00	0.00	0.03	0.29	0.01	0.36	0.00	0.31	0.00
r	0.00	0.00	0.01	0.02	0.16	0.42	0.00	0.31	0.08

Table S12. Comparison of amino acid chirality pattern versus structural digit chirality pattern for predicted top structures of the entire sequence space (3,375 sequences) at position R4.

Position R4

Structural digits (columns) vs. Amino acids (rows); colour scale: Fraction (0.0–1.0)

Amino acids	Β	Π	Γ	Λ	β	π	γ	λ	δ
A	0.01	0.48	0.04	0.47	0.00	0.00	0.00	0.00	0.00
V	0.00	0.71	0.00	0.29	0.00	0.00	0.00	0.00	0.00
F	0.00	0.60	0.00	0.40	0.00	0.00	0.00	0.00	0.00
N	0.00	0.32	0.07	0.45	0.00	0.00	0.00	0.16	0.00
S	0.02	0.27	0.00	0.71	0.00	0.00	0.00	0.00	0.00
D	0.00	0.32	0.13	0.54	0.00	0.00	0.00	0.01	0.00
R	0.00	0.44	0.00	0.56	0.00	0.00	0.00	0.00	0.00
G	0.00	0.02	0.00	0.44	0.06	0.12	0.00	0.36	0.00
a	0.00	0.00	0.00	0.17	0.01	0.18	0.00	0.63	0.01
v	0.00	0.00	0.00	0.04	0.00	0.02	0.00	0.85	0.09
f	0.00	0.00	0.00	0.14	0.00	0.16	0.00	0.67	0.03
n	0.00	0.00	0.00	0.39	0.00	0.12	0.00	0.46	0.03
s	0.00	0.00	0.00	0.13	0.01	0.25	0.00	0.61	0.00
d	0.00	0.00	0.00	0.25	0.00	0.04	0.00	0.71	0.00
r	0.00	0.00	0.00	0.28	0.01	0.11	0.00	0.60	0.00
